# The Contribution of Foods and Beverages of Low Nutritional Value to the Diets of Swedish Adolescents, by Food Group, Time and Place. A Nationally Representative Study

**DOI:** 10.3390/nu13072450

**Published:** 2021-07-17

**Authors:** Anna Karin Lindroos, Lotta Moraeus, Jessica Petrelius Sipinen, Eva Warensjö Lemming, Emma Patterson

**Affiliations:** 1 Department of Risk and Benefit Assessment, Swedish Food Agency, 751 26 Uppsala, Sweden; lotta.moraeus@slv.se (L.M.); jessica.petreliussipinen@slv.se (J.P.S.); eva.warensjolemming@slv.se (E.W.L.); emma.patterson@slv.se (E.P.); 2 Department of Internal Medicine, the Sahlgrenska Academy, University of Gothenburg, 405 30 Gothenburg, Sweden; 3 Department of Global Public Health, Karolinska Institutet, 113 65 Stockholm, Sweden

**Keywords:** discretionary foods, energy dense foods, children, eating out-of-home, biomarkers, nutritional status, 24-h recall, cross-sectional

## Abstract

Adolescence is a time in life when lifestyle behaviours are acquired. One indicator of poor diet quality is the intake of foods and beverages with a relatively low nutritional value. Using the Australian classification of such foods, termed “discretionary”, we classified the intakes of Swedish adolescents who participated in the Riksmaten Adolescent 2016–17 national dietary survey. From selected schools, 3099 adolescents in age groups 11–12, 14–15 and 17–18 years provided two 24-h recalls. Intakes and healthy dietary scores were calculated. Plasma ferritin, folate and 25(OH)D were available for a third. Almost 40% of total energy came from discretionary foods/beverages. Adolescents with higher intakes were more likely to be female, older, from a low socioeconomic position-household and born in Sweden. Most discretionary foods/beverages were consumed on weekend days and during in-between meals, outside of the home and at school. Percent energy from discretionary intake was associated with healthy dietary scores but not nutritional status. A substantial amount of energy was obtained from discretionary foods/beverages, and we found that consumption is pervasive across sociodemographic factors, time and place. Addressing this pattern will require a comprehensive approach to food environments and behaviours to reach all adolescents in an equitable manner.

## 1. Introduction

Poor dietary habits are associated with increased risk of diseases such as type 2 diabetes, cardiovascular diseases, certain cancers and overweight/obesity [1]. Food habits are formed early in life and dietary behaviours track into adulthood [2]. Adolescence is a particularly vulnerable time in life, and although dependence on the family environment is still strong, peers and the environment outside the home become more important [3]. During this transition period in life, key lifestyle behaviours are acquired [4].

Several studies have investigated the diet of children and adolescents and have concluded that their diets overall fail to meet dietary guidelines and that adolescents consume too little vegetables, fruits and milk/dairy products and too much meat/meat products, fats and sweets [5,6,7]. In addition, diet quality is poorer in groups with lower socioeconomic position [8]. In Sweden, results from the latest dietary survey of adolescents are similar with a low consumption of fruit, vegetables and whole grains and a high consumption of meat/meat products (particularly in boys) [9] and added sugars [10] compared to recommendations. Furthermore, participants in households with lower education levels were found to score lower on two different healthy eating indices adapted for adolescents and the Swedish dietary guidelines [11]. One important indicator of poor diet quality is the intake of foods and beverages with a relatively low nutritional value, i.e., foods high in energy and nutrients that the general population consume more than the recommended amounts of. These are foods typically high in sugar, salt and/or saturated fat such as confectionary, cakes, biscuits, desserts, ice cream, salty snacks and sugar sweetened beverages (SSBs), but may also include foods such as hamburgers, fries, pizza and highly processed foods. There is, however, no consensus on how to define foods and beverages with a low nutritional value. Commonly occurring terms in the literature can refer to their nutritional composition (e.g., “SoFAS”, “energy dense”, “low nutritional value”), their level of processing (e.g., “ultra-processed foods”) or their presumed place—or lack of—in the diet (e.g., “unhealthy”, “junk foods”).

In Sweden, the dietary recommendation is to limit foods high in added sugars and salt as well as red and processed meats. However, apart from the most obvious foods such as sugar-sweetened beverages, sweets, ice cream, cakes and crisps, these foods are not well defined. In contrast, the Australian Dietary Guidelines define discretionary foods as foods and drinks that do not fit into the five main food groups of fruit, cereals, meat and meat alternatives, dairy and vegetables and legumes/beans [12]. The discretionary food choices are thus foods and drinks that “are not necessary part of a healthy diet and are high in saturated fat, added sugars, salt and/or alcohol” [13]. Based on this division of foods, a classification system of discretionary foods has been developed [14] that has mainly been used in the research of children and adults in Australia, e.g., [15,16,17], but also in studies of children and adolescents in the UK [18,19,20]. As we lacked a definition, we looked at others (e.g., level of ultra-processing) but felt that the Australian definition was very compatible. As it had already been successfully applied in other European settings, we chose to use this one.

The contribution to total energy intake from discretionary foods varies depending on age, classification and setting, and figures between 26% and 43% have been reported in children and adolescents [15,16,19,21,22]. A high intake of discretionary foods and beverages is also associated with a lower total intake of micronutrients [23]. Iron deficiency anaemia is common in teenaged girls [24] and low status of vitamin D and folate is a nutritional concern among adolescents in Europe [5]. Whether a high intake of discretionary foods and beverages as a marker of poor diet quality is related to more objective measures of nutritional status, such as biomarkers for iron, vitamin D and folate, has, to our knowledge, not been reported.

In order to guide and formulate public health policy strategies to improve adolescent’s diets equitably, more knowledge on the factors—sociodemographic, temporal, locational, environmental—associated with consumption of discretionary foods and beverages is needed. Intake of discretionary foods also vary depending on country and geographical area [8], making country-specific analyses necessary. Sweden, where free school lunches are offered to all children up to the age of 16 and to most students in high school, constitutes a unique setting to study. So far there are no in-depth analyses of discretionary food consumption using nationally representative data from Swedish adolescents. The aim of this study is therefore to describe the intake of foods and beverages with low nutritional value, using the Australian classification of discretionary foods, in the Swedish national dietary survey Riksmaten Adolescents 2016–17 to see if differences in location, meal type, day of week and population subgroup emerge. Furthermore, we explore differences in overweight status, two diet quality scores and the biomarkers of plasma folate, ferritin and 25(OH)D in a subgroup by intake of discretionary foods and beverages.

## 2. Materials and Methods

### 2.1. Study Design

Data were derived from the Swedish national dietary survey Riksmaten adolescents 2016–17, a cross-sectional dietary survey of school children in grades 5 (11–12 years old), 8 (14–15 years old) and grade 11 (17–18 years) carried out by the Swedish Food Agency (SFA), Uppsala, Sweden between September 2016 and May 2017. Diet was assessed on two independent days using a web-based, self-administered 24-h recall method and information on background, lifestyle and frequencies of foods were collected through questionnaires on the web. Weight and height were measured by trained staff at a school visit and non-fasting blood samples were drawn from a subsample. A detailed description of the survey and the methods has been published elsewhere.

### 2.2. Population an, d Sampling

A total of 619 schools were randomly selected from the Swedish school registry by Statistics Sweden to represent pupils in the three age groups across Sweden. Schools were sampled based on type of municipality, whether it was a public or charter school (independent school with public funding) and geographical spread. Forty percent of schools were randomly selected for blood and urine sampling. Of the 619 schools, 18 schools were excluded as they had too few pupils or only provided language introduction classes, leaving 601 schools to be invited. In total, 131 schools accepted and pupils in 1 or 2 classes were selected to take part. Altogether, 5145 pupils were invited, and 3477 pupils participated in some stage of the survey. Drop-out analyses show that the participating pupils were overall representative of the population with regard to socioeconomic background and school type. All types of municipalities were represented, and the geographical spread corresponded well to the underlying population [25].

The 3099 pupils with complete information on diet (see Dietary assessment) formed the main study group. A subsample, 2377 of the 5145 pupils, were also asked to provide blood and urine. The 1105 pupils with complete information on diet and blood constitute the study subgroup for the biomarker analyses in the present analysis.

### 2.3. Dietary Assessment

The participants reported their dietary intake in the validated, web-based dietary assessment method RiksmatenFlex [26]. All foods and beverages were recorded retrospectively, as a 24-h recall, on two non-consecutive days. The first diet day was the day prior to the school visit; the second diet day was automatically generated by the system to get an even distribution of days over the week at group level. Both days were required and 3099 provided this.

The participants could search foods from a list of 778 foods and portion sizes were specified using a picture portion guide. Time, meal type (breakfast, lunch, dinner/supper, snack, other eating, drink only) and place (at home, at school, in a restaurant/bar/café, at an event e.g., cinema/theatre/sports, in someone else’s home, at street food/convenience stores, other place including work, or while traveling) of an eating occasion was also recorded. “At work” was grouped with “other” because of the limited number of participants reporting an intake at work. Type and place were self-defined by the participant. Automatic reminders were generated if drinks were not included in a meal and if less than four foods were recorded in a main meal. The complete day was finally reviewed by the participant and a list of foods easily forgotten (i.e., bread, fat spread, soft drinks, crisps, fruit, ketchup) was generated to help the participants to remember all their intakes. The food list was linked to the Swedish national food composition database (version Riksmaten Adolescents 2016–17) allowing for direct calculation of energy, nutrients and foods intake per day, place or eating occasion depending on the research question. Added sugars were calculated according to the method described in Wanselius et al. [10].

### 2.4. Definition of Discretionary Foods

Each food in the food list was classified as discretionary or not using the Australian classification system [14]. Our database contained 778 foods in 110 food groups, each with between 1 and 46 foods. Most foods could be easily classified as corresponding to one of the five Australian non-discretionary food groups: (1) Bread, cereals, rice, pasta and noodles; (2) vegetables; (3) fruits; (4) dairy products and (5) meat and fish. The classification of many discretionary foods was also straightforward, for example all sugar-sweetened beverages (SSBs), all sweets, all cakes, etc. For some foods and mixed dishes, however, the classification had to be done at the food level. For example, breaded and deep-fried fish dishes were coded as discretionary whereas other fish dishes were not. Meat was coded as discretionary if it was processed, and sandwiches and cereal-based mixed dishes were coded as discretionary if saturated fat was >5 g per 100 g. Because of differences in the national food databases, for some foods the classification was challenging and when in doubt the discretionary classification of Australian foods was consulted [27]. In total, 273 of the 778 foods in our database were classified as discretionary (35%). Some groups such as “Pizzas, pies, sandwiches” were broad and contained both discretionary and non-discretionary foods. Where the food group is presented below only the discretionary foods are included. For ease of interpretation, and as described in Supplementary File S1, a few of the original 110 food groups that were predominantly discretionary were combined. For example, the three groups chocolate, sweets miscellaneous and sweets excluding chocolate were combined to Sweets and chocolate. The other combined groups were desserts and ice cream, SSBs, and cakes and biscuits. The full classification of foods and beverages in the Riksmaten Adolescence food database according to discretionary status, including the rationale, is available as Supplementary File S1.

### 2.5. The Eating Index Scores SHEIA15 and RADDS

In order to capture a healthy dietary pattern, two different healthy eating scores were used that were developed for this population [11]. The Swedish Healthy Eating Index for Adolescents 2015 (SHEIA15) is based on the 2015 Swedish food-based dietary guidelines (mentioning fruit and vegetables, seafood, wholegrains, healthy fats, low fat dairy, processed meat and sugar). The Riksmaten Adolescents Diet Diversity Score is a measure of the diversity aspect of the dietary guidelines. See Moreaus et al. [11] for more details.

### 2.6. Data from Questionnaires

Information on age and sex was collected from class lists. Education, as a measure of socioeconomic position, was reported in an online questionnaire completed by the participants’ parents. The parents reported their own and their partner’s educational level and the highest education achieved in the household was used in the analyses. The five response levels of education were collapsed into ≤12 years and >12 years. Participants were classified as being born in Sweden or not.

### 2.7. Anthropometry and Blood Sampling

Weight and height were measured by field staff at a school visit using standardised methods and portable equipment. Weight was measured to the nearest 0·1 kg using SECA 862 or 899 digital weighing scales. Height was measured to the nearest 0·1 cm using SECA 213 portable stadiometers. BMI was calculated (kg/m^2^), and weight status was determined using the International Obesity Task Force reference [28]. BMI standard deviation score (sds) was calculated using the WHO reference [29].

Non-fasting blood was drawn by trained staff. The blood was spun, and the samples were temporarily stored at −20 °C before they were transported for long-term storage in −80 °C freezers.

### 2.8. Biomarkers of Nutritional Status

Plasma folate, ferritin and CRP (C-reactive protein) were analysed on an Abbott Architect ci8200 system (Abbott Laboratories, Abbott Park, IL, USA) at the Department of Clinical Chemistry and Pharmacology, University of Uppsala and the Academic hospital, Uppsala, Sweden. The laboratory is certified according to SS-EN ISO/IEC 15189. The analytical uncertainty (coefficient of variation) for the methods were: folate (chemiluminescense) 7–8%, ferritin 5% and CRP (turbidimetri) 4–5%. Ferritin concentrations of participants with a CRP ≥ 5 (*n* = 48) were not used in the analyses in order to avoid false high ferritin values due to an on-going infection. Total plasma 25-hydroxyvitamin D [25(OH)D] was calculated by summing plasma 25(OH)D3 and 25(OH)D2, which had been determined with HPLC atmospheric pressure chemical ionization (APCI) mass spectrometry (MS) at Vitas, Oslo, Norway (www.vitas.no 16 July 2021) as previously described [30].

### 2.9. Statistics

For the descriptive analyses of intakes by days of the week, location and meal type, the population proportion method was used [31]. For other analyses, proportions were calculated at the individual level. *t*-tests were performed to check for differences in percent discretionary energy intake between subgroups, and multiple linear regression was performed to investigate if the observed differences held when controlling for other predictors. To investigate the association between percent discretionary energy intake and nutritional biomarkers and dietary scores, linear regression was performed. The significance level was set at *p* < 0.05. All analysis was performed using Stata v15 [32].

## 3. Results

### 3.1. The Study Population and Mean Intakes of Energy and Macronutrients

The study population consisted of 3099 children and adolescents and is described in Table 1. The population mean intakes of energy and macronutrients is provided for all food and beverage intake and for discretionary food and beverage intake. The proportion of energy from discretionary foods and beverages was quite consistent across the age and gender subgroups; between 34 and 39%. Unsurprisingly, the majority of added sugar (82–87% of the total, depending on subgroup) came from discretionary foods/beverages. Almost half of saturated fat (45–50% of the total) came from discretionary foods/beverages.

With regards to sociodemographic characteristics, the intake of energy from discretionary foods and beverages increased with age, was slightly higher in girls, in adolescents born in Sweden, and in adolescents from households with lower socioeconomic position (SEP) (Table 2). Although the differences are statistically significant, this is likely in part due to the large sample size. All subgroups obtained a substantial amount of energy from discretionary foods and beverages. A multiple linear regression confirmed that each of these remained significant predictors even when the other factors were held constant (*p* < 0.001 for all factors).

### 3.2. What Are the Main Discretionary Foods Consumed?

The main food groups contributing to the intake of discretionary intake, by energy, are presented in Table 3. (See Supplementary Table S1 for contributions by weight). Certain food groups appeared consistently in all age and gender subgroups. Sweets and chocolate, pies, pizza and sandwiches, cakes and biscuits, and SSBs are among the top four (by energy) for almost all. By energy, sweets and chocolate contributed 8.6–21.4% of the total and was the group that contributed most to four of the six subgroups. SSBs and pies, pizza and sandwiches are among the top three (by weight) for all, with the third group being sweets and chocolate in grades five and eight, replaced by “Light” (artificially sweetened) drinks for girls in grade 11, and beer for boys in grade 11. Other foods also appeared consistently in the top ten (by weight), such as desserts and ice cream, cakes and biscuits, and sausages, while others appeared only in certain subgroups, such as beer (boys in grade 11). SSBs contributed to almost half of all discretionary intake by weight in all subgroups (40.9–46.9%).

### 3.3. Where Are Most Discretionary Foods Consumed?

**Figure 1:**
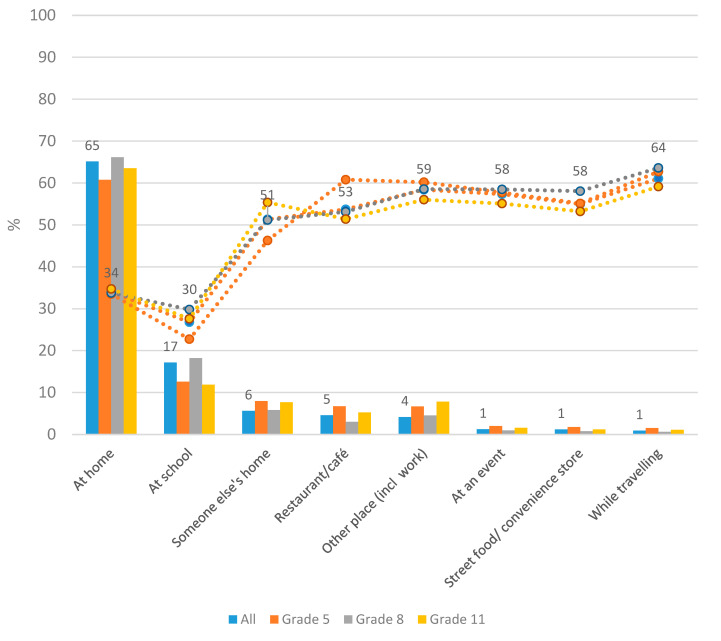
Food/beverage consumption by location. Number of food/beverages consumed, expressed as a proportion of total energy, at each location (**bars**), and number of food/beverages consumed at that location that are discretionary, expressed as a proportion of all energy consumed at that location (**lines**). Provided data labels are for “All”.

The majority, 65%, of all food and beverage consumption occurred at home (Figure 1). The next most common locations were school (17%) and someone else’s home (6%), and the pattern was similar in all age groups. The proportion of energy intake that came from discretionary foods/beverages was greatest while traveling, at other places (including at work), at street food/convenience stores and at events. However, these locations did not contribute much to overall intake. Conversely, although home and school were locations where the least energy from discretionary foods/beverages was consumed, they were still locations where most of the total intake occurred.

### 3.4. When Are Most Discretionary Foods Consumed?

With respect to meals, breakfast was the meal where both the least energy was consumed (17–20% of total energy) and least discretionary foods/beverages were consumed as a proportion of total energy from that meal type (Table 4). This was very consistent across age and gender subgroups (range: 19–20%), see Supplementary Table S2. Dinner, lunch and snacks between meals were relatively comparable, with 28–36% energy coming from discretionary foods (Table 4). The meal type where the greatest proportion of energy came from discretionary foods/beverages was the meal type classified as “Other” (range: 79–84%, Table S2). In the eldest age group, the meal type consisting of “Beverage (mainly)” also consisted of predominantly discretionary energy (85%, Table S2).

**Figure 2:**
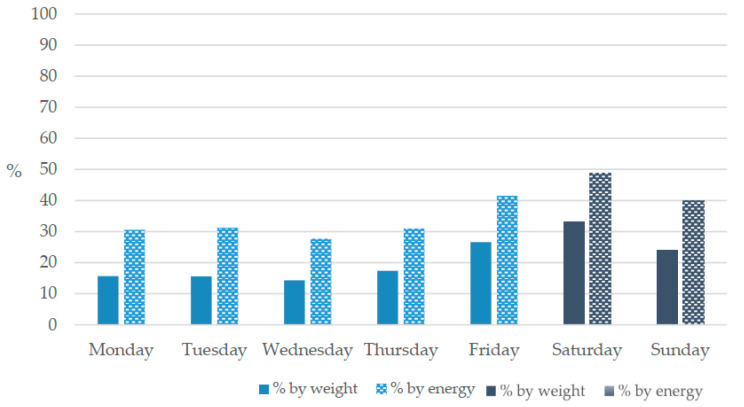
Discretionary food/beverage intake as a proportion of total intake by day of week.

Regarding day of the week, Saturday was the day on which most discretionary foods/beverages were consumed, followed by Friday and Sunday (Figure 2). Lower and similar consumption was seen on Monday to Thursday. Between 40% and 50% of all energy intake on weekend days came from discretionary foods/beverages, and this was consistent across age and gender subgroups.

### 3.5. Nutritional Status and Healthy Diet Scores according to Consumption Levels of Discretionary Foods/Beverages

The values for plasma folate, ferritin and vitamin D are presented in Table 5, according to quartile of discretionary food/beverage intake. No clear pattern was seen with quartiles of discretionary intake. However, there was a clear association in all gender and age groups between quartile of discretionary intake and healthy dietary scores.

## 4. Discussion

This cross-sectional study investigated adolescents’ intake of foods high in saturated fat, salt and added sugar using the Australian discretionary food classification. The results show that the contribution of discretionary foods to total energy intake was high, almost 40 percent. The results are in line with studies on children and adolescents in Australia [16,33] and the UK [19] using the Australian discretionary foods classification and in the US using a different classification, SoFAS (Solid fats and added sugars) [22]. The energy from discretionary foods and beverages increased by age and was highest in 17–18-year-old boys, however the contribution of discretionary foods to total energy intake was fairly similar across the age groups (around 36%) with the exception of 11–12-year-old boys (34%) and 17–18-year-old girls (39%). A small increase in the percentage of discretionary food energy from the youngest age group (11–12 years) to the mid age group (13–15 years) and a higher energy percentage from girls than boys was also noted in the UK study [19].

The food groups sweets and chocolates (5.7%), pies, pizza and sandwiches (5.3%), SSBs (3.9%), cakes and biscuits (3.6%), and crisps and savoury snacks (2.3%) were the top contributors to energy intake in all age groups, and combined they contributed to over 20% of the total energy intake. Differences between age and sex in our study were small but the contribution of energy from sausages dropped in girls in the two older age groups, whereas the contribution of Sweets and chocolates was highest in these two groups. Similar food groups were top contributors to the discretionary energy intake in the UK [19], Australia [33] and the US [22] but direct comparison is difficult as foods are grouped differently. Results were very similar to a previous study of Swedish children and adolescents from 1998–9, where SSBs, sweets and chocolate, chips and crisps, cakes and biscuits and other sweet foods together accounted for almost one-fifth of energy and saturated fat and two-thirds of sucrose intakes [34].

However, sugar-sweetened beverages (SSBs) are among the top contributors to discretionary energy in all cited studies [16,19,22,33]. SSBs contribute to energy intake but do not contain any other nutrients. Several studies have shown that a high consumption of SSB is associated with a poorer diet quality [23,35], but a high consumption of SSBs could also be a marker of poor nutrient quality of the rest of the diet [36]. Consumption of SSBs is also associated with increased risk of overweight and obesity [37] and thus an important target for dietary intervention.

The proportion of energy from discretionary foods at places outside the home and the school was high in our study and varied between 51% and 64%. Similar figures have been reported before [19]. Other studies have also reported that foods eaten outside the home are less healthy than at foods eaten at home [38,39]. Around one third of the energy at home and almost a third of the energy at school came from discretionary foods. This is similar to results reported on discretionary food energy at home but the proportion of discretionary energy at school is lower in our study compared to the UK study [19]. This difference could partly be explained by the Swedish school meal system. Schools by law are required to serve free and nutritious school lunches to all pupils up to grade 9, and in fact pupils may not choose to bring a lunch with them from home. Fried food and desserts are not a feature of the Swedish school lunch, and lunches are often healthier than meals consumed outside of school [40]. Despite this, almost a third of the energy at school came from discretionary foods and beverages, highlighting that both school lunches and the entire school environment have to be targeted in order to improve the dietary intake of school children. Many pupils are allowed to leave the school grounds during lunch and some do skip the school lunch, so pupils may consume discretionary foods/beverages outside of lunch time, either bought at a school cafeteria or brought from outside. In fact, our results highlight the importance of providing healthy food choices at all places where adolescents eat. Although the proportion of discretionary food energy was highest at places outside the home and school, it should be stressed that most foods and beverages were consumed at home and thus the largest amounts of discretionary foods and beverages were also consumed at home. Although the proportion of energy intake from discretionary foods and drinks at school were lower than in other out-of-home places, the school is still a place where many discretionary foods are eaten. Thus, public health interventions aiming at improving the diet quality of children and adolescents need to target both the home and school environment, as well as the food environment at places outside the home and school.

There was also a large difference in intake of discretionary foods by day of the week. Almost half of the total energy intake came from discretionary foods on the weekend days and Fridays compared to around one third during the other weekdays. A poorer diet quality during weekend days compared to weekdays has been reported in children [41] and adolescents [42], but the differences were less pronounced in Danish adults [42]. In Sweden, children are often encouraged to consume sweets on just one day of the week (“Saturday sweets”), whether this leads to an exaggerated association between less healthy food and the weekend than in countries where this is less of a tradition has not to our knowledge been explored. The proportion of energy from discretionary foods differed by meal type. At breakfast, one fifth of the energy came from discretionary foods, the lowest amount of all meals. Several studies have reported that eating breakfast is associated with a higher nutrient intake [43,44] and that the foods eaten at breakfast contribute to a higher nutrient intake [45]. In contrast, around 80% of the energy at the meals the participants defined as “Other” came from discretionary foods, whereas in the meals defined as snacks around 30% of the energy came from discretionary foods. Although the Swedish word for a snack literally means “between meals”, it tends to refer to healthier or more substantial small meals (e.g., a piece of fruit, or a sandwich with milk), rather than all eating between main meals (biscuits, crisps, sweets, etc). Hence many unhealthy foods outside of main meals were registered as “Other” rather than “Snacks”, although some overlap between these two meal types also occurred, as all meals were self-defined by the participants. Meals are defined very differently across studies. In some studies meals are predefined, whereas in other studies, like this one, participants define time and meal types themselves. Direct comparison between studies is therefore difficult, but in general discretionary foods provide a higher proportion of the energy in snack/in between meals than in main meals [33].

With increasing healthy eating index scores of the SHEIA15 and RADDS, the intake of discretionary foods decreased. This suggests that the intake of discretionary foods is related to an overall healthy eating pattern. We have previously reported that almost one third of the two older age groups of girls had low plasma ferritin levels (<15 µg/L) and that between 10–15% of the participants had 25(OH)D below 30 nmol/L, indicating vitamin D deficiency. Between 7–26% had also low folate levels (<6.8 nmol/L) [46]. However, we found no clear pattern between the intake of discretionary foods and the nutritional biomarkers of folate, ferritin and 25(OH)D. It is possible that the food sources of the nutrients folate, iron and vitamin D are more important for the levels of these biomarkers than the consumption of discretionary foods. Weak associations between folate intake and overall healthy eating and plasma folate [43] and vitamin D intake and 25(OH)D [47] have also been reported. On the other hand, iron and vitamin D status are also influenced by non-dietary factors [48]. Moreover, the intake of discretionary foods was not associated with overweight and obesity. This is not surprising as cross-sectional studies rarely find associations between unhealthy eating and weight status and in some studies even negative associations [49]. Even if they do find positive associations, reverse causation cannotbe ruled out. Thus, discretionary food intake does not appear to be a great indicator of the status of these nutrients but may be more suited as a marker of a healthy dietary pattern. In-depth analyses of the factors associated with intakes and nutritional status of these three nutrients, as well as added sugars, is on-going.

All subgroups, regardless of age, gender and SEP, obtain a substantial amount of energy from discretionary foods and beverages. This points to the importance of a health-promoting food environment throughout the life course and in all locations where food is available. Differences in SEP found in this study may warrant further attention but can also be seen to primarily reinforce the fact that health-promoting initiatives must work for all sectors of society. Certain foods/beverages are obvious sources of discretionary energy (e.g., SSBs) and are commonly singled out for action, but they are by no means the only sources in the diets of Swedish adolescents. Initiatives focusing on SSBs—such as taxes coupled to sugar levels—have been shown to be successful in other countries and should be explored in Sweden [50]. Other kinds of instruments such as increased subventions for healthier foods, enhanced front-of-pack labelling, voluntary agreements with industry, etc. may also need to be explored. Given the ubiquitousness of these foods/beverages, an array of instruments is likely to be required, tailored to the local context. All involved in the upbringing of children, at all levels from governments and policy makers to parents, need to join forces to provide a healthy environment for children to grow up in.

### Strengths and Limitations

There is no consensus on how to define foods of relatively low nutritive value. Although nutritional needs vary according to age and health status, the general adult population in high-income countries tends to be overweight and have a high intake of sugar, salt and saturated fat. Despite this, pinning down the foods that are rich sources of these nutrients using a workable and transparent definition remains elusive. A wide range of terms has been used in the literature, making comparisons with other studies difficult.

The lack of detail may have both led to an over- or underestimation of discretionary intake. It is a challenge when using a definition defined for another database. Our database contained fewer and more composite/generic foods compared to the Australian food composition database (778 and 1534 foods, respectively). When a definition is developed for another population it can also be a problem. For example, sweetened yoghurt and milk were not classed as “discretionary”, even though this is not entirely in line with current Swedish food-based dietary guidelines, where low-fat, unsweetened versions are recommended. But on the whole, it was easy to apply the Australian definition as the food culture is broadly similar, being a high-income country with a lot of similarities to western Europe. The Australian definition has also been successfully used in other populations.

This study was of cross-sectional design, so it is not possible to draw conclusions about the temporality of associations between dietary intake and nutritional status. All data were self-reported by the participants, and meal types and locations were self-defined. We have examined energy-underreporting in the Riksmaten Adolescent study. As with most self-reported dietary data, we found evidence of under- and over-reporting of both unhealthy foods and healthy foods, but at group level, the level of energy reporting was found to be very plausible [46]. Riksmaten Adolescents is the only national representative survey of Swedish adolescents conducted to date and the method used was valid. A further strength is that objective measures of nutritional status were available for a subgroup.

## 5. Conclusions

We did not find associations between discretionary intake and the nutritional biomarkers available, and discretionary intake may not be a good indicator of the status of these particular nutrients. It was, however, correlated to other aspects of dietary quality, such as diversity and overall health scores. A substantial amount of energy is obtained from discretionary foods/beverages across sociodemographic factors, time and place. While this analysis gives some indications about where and when these foods are being consumed, and by whom, it is also clear that the pervasive nature means that addressing this pattern will require a comprehensive approach to food environments and behaviours to reach all adolescents in an equitable manner.

## Figures and Tables

**Table 1:** Characteristics of participants and mean intakes of total energy and macronutrients from all foods/beverages and from discretionary foods/beverages.

	School Grade: 5th						8th						11th					
	Girls	(*N* = 599)		Boys	(*N* = 490)		Girls	(*N* = 574)		Boys	(*N* = 476)		Girls	(*N* = 577)		Boys	(*N* = 423)	
Age (mean, sd)	11.5	0.4		11.5	0.4		14.5	0.4		14.5	0.4		17.7	0.6		17.7	0.6	
High SEP, *N* (%)	351	62.8		285	58.2		347	60.5		260	54.6		298	51.6		217	51.3	
Born in Sweden, *N* (%)	517	92.5		442	90.2		515	89.7		408	85.7		507	87.9		359	84.9	
Overweight/obese, *N* (%)	125	22.4		104	21.2		101	17.6		80	16.8		127	22.0		109	25.8	
All foods/beverages	Mean	SD		Mean	SD		Mean	SD		Mean	SD		Mean	SD		Mean	SD	
Weight, g	1990	594		2109	743		2114	655		2599	947		2303	788		2872	1008	
Energy (MJ)	7.8	2.7		8.4	3.1		8.0	2.8		10.7	4.3		8.2	2.7		10.9	3.9	
Protein, g	75	26		86	33		74	26		108	45		73	26		116	52	
Total fat, g	72	32		78	37		76	33		100	47		79	33		103	42	
Saturated fat, g	29	14		31	16		30	15		40	20		30	13		40	18	
Carbohydrates, g	218	79		230	92		221	83		292	132		225	79		287	115	
Added sugar, g	61	43		49	36		66	45		52	38		67	46		51	41	
Fibre, g	16	6		17	7		17	8		20	10		18	9		20	10	
…of which discretionary foods/beverages (%)																		
	Mean	SD	%	Mean	SD	%	Mean	SD	%	Mean	SD	%	Mean	SD	%	Mean	SD	%
Weight, g	376	293	19	417	368	20	387	287	18	555	427	21	487	404	21	690	588	24
Energy (MJ)	2.8	2.1	36	2.8	2.3	34	2.9	2.1	37	3.8	3.0	36	3.2	2.1	39	4.0	2.8	37
Protein, g	15	13	20	16	16	19	15	14	20	21	21	19	15	13	21	25	28	22
Total fat, g	35	27	49	34	31	44	36	27	47	45	36	45	38	27	48	45	33	44
Saturated fat, g	14	12	48	14	14	45	15	12	50	19	15	48	15	11	50	18	14	45
Carbohydrates, g	73	62	33	75	71	33	78	65	35	104	104	36	84	63	37	104	87	36
Added sugar, g	51	42	84	39	34	80	57	43	86	43	37	83	58	45	87	42	39	82
Fibre, g	3	3	19	3	4	18	3	4	18	4	5	20	4	4	22	4	5	20

**Table 2:** Discretionary intake according to sociodemographic factors.

	*N*	% of Total E that Is Disc.	*p* *
SEP			<0.001
Low	1341	37.7	
High	1758	35.5	
Grade			<0.001
5th	1049	34.9	
8th	1050	36.5	
11th	1000	37.8	
Gender			0.001
Girls	1710	37.4	
Boys	1389	35.5	
Country of birth			0.007
Sweden	2748	36.7	
Not Sweden	337	34.0	

* Two-sided *t*-test; SEP, socioeconomic position; E, energy; Disc, discretionary.

**Table 3:** Contribution of discretionary foods/beverages to total intake of all foods/beverages and to total discretionary intake, by energy.

	All (*N* = 3099)				
Foods from Food Group	% of Disc. E	% of Total E			
Sweets, chocolate	15.7	5.7			
Pies, pizza, sandwiches *	14.5	5.3			
Sugar-sweetened beverages	10.8	3.9			
Cakes, biscuits	10.0	3.6			
Crisps, savoury snacks	6.2	2.3			
Sausages *	5.7	2.1			
Sauces, dressings *	5.0	1.8			
Potato products and dishes *	4.4	1.6			
Mixed fat spread *	4.3	1.5			
Desserts, ice cream	4.2	1.5			
School grade: 5th					
Girls (*N* = 599)			Boys (*N* = 490)		
	% of disc. E	% of total E		% of disc. E	% of total E
Sweets, chocolate	16.5	5.9	Pies, pizza, sandwiches *	14.5	4.9
Pies, pizza, sandwiches *	11.8	4.2	Sweets, chocolate	13.6	4.6
Cakes, biscuits	11.0	3.9	Sugar-sweetened beverages	11.0	3.7
Sugar-sweetened beverages	9.1	3.3	Sausages *	8.8	3.0
Sausages *	6.7	2.4	Cakes, biscuits	7.4	2.5
Crisps, savoury snacks	5.6	2.0	Crisps, savoury snacks	6.3	2.1
Desserts, ice cream	5.1	1.9	Potato products and dishes *	5.0	1.7
Mixed fat spread *	5.1	1.8	Desserts, ice cream	4.3	1.4
Potato products and dishes *	4.8	1.7	Mixed fat spread *	4.3	1.4
Sauces, dressings *	4.3	1.6	Sausage dishes	4.0	1.3
8th					
Girls (*N* = 574)			Boys (*N* = 476)		
	% of disc. E	% of total E		% of disc. E	% of total E
Sweets, chocolate	21.4	7.9	Sweets, chocolate	18.1	6.5
Cakes, biscuits	13.0	4.8	Pies, pizza, sandwiches *	16.6	6.0
Pies, pizza, sandwiches *	12.4	4.6	Sugar-sweetened beverages	11.3	4.1
Sugar-sweetened beverages	8.9	3.3	Cakes, biscuits	8.2	2.9
Crisps, savoury snacks	5.7	2.1	Crisps, savoury snacks	6.4	2.3
Desserts, ice cream	4.6	1.7	Sausages *	5.3	1.9
Sauces, dressings *	4.6	1.7	Potato products and dishes *	4.9	1.8
Mixed fat spread *	4.6	1.7	Sausage dishes	4.6	1.6
Sausage dishes	4.0	1.5	Sauces, dressings *	4.3	1.5
Sausages *	3.8	1.4	Mixed fat spread *	3.6	1.3
11th					
Girls (*N* = 577)			Boys (*N* = 423)		
	% of disc. E	% of total E		% of disc. E	% of total E
Sweets, chocolate	15.5	6.1	Pies, pizza, sandwiches *	17.0	6.2
Pies, pizza, sandwiches *	14.2	5.5	Sugar-sweetened beverages	13.6	5.0
Cakes, biscuits	12.3	4.8	Sweets, chocolate	8.6	3.2
Sugar-sweetened beverages	10.9	4.3	Cakes, biscuits	7.5	2.7
Sauces, dressings *	7.4	2.9	Crisps, savoury snacks	7.0	2.6
Crisps, savoury snacks	6.2	2.4	Sausages	6.4	2.3
Mixed fat spread *	4.8	1.9	Sauces, dressings *	5.1	1.9
Desserts, ice cream	4.5	1.8	Potato products and dishes *	4.9	1.8
Sausages	3.9	1.5	Desserts, ice cream	3.5	1.3
Potato products and dishes *	3.8	1.5	Mixed fat spread *	3.3	1.2

* A food group that contains both discretionary and non-discretionary foods, but only the discretionary foods are included here. E, energy; Disc, discretionary.

**Table 4:** Contribution of meal types to consumption of total energy, the proportion of each meal (by energy) that is from discretionary foods/beverages, and the top three discretionary foods/beverages contributing most to total energy at each meal type.

	All (*N* = 3099)	
	% of Total E	% of Which Is Disc. E
Breakfast	18.7	19
Pies, pizza, sandwiches *	0.9	
Mixed fat spread *	0.8	
Cakes, biscuits	0.3	
Lunch	25.2	28
Pies, pizza, sandwiches *	1.2	
Sausages *	0.8	
Sugar-sweetened beverages	0.7	
Dinner	32.4	33
Pies, pizza, sandwiches *	2.5	
Sugar-sweetened beverages	1.6	
Potato products and dishes *	1.0	
Snack/between meal	9.2	36
Cakes, biscuits	0.9	
Sweets, chocolate	0.4	
Pies, pizza, sandwiches *	0.4	
Other	13.3	82
Sweets, chocolate	4.7	
Cakes, biscuits	2.0	
Crisps, savoury snacks	1.8	
Beverage (mainly)	1.2	72
Sugar-sweetened beverages	0.4	
Cider, alcopops	0.2	
Beer	0.1	

* A food group that contains both discretionary and non-discretionary foods, but only the discretionary foods are included here. E, energy; Disc, discretionary

**Table 5:** Concentrations of plasma folate, ferritin and 25(OH)D in a subpopulation, weight status and healthy diet scores in the whole population, according to quartile of discretionary foods/beverages by energy.

	School Grade: 5th								8th								11th							
	Girls				Boys				Girls				Boys				Girls				Boys			
Biomarkers *	*N*	mean	sd	P	*N*	mean	sd	P	*N*	mean	sd	P	*N*	mean	sd	P	*N*	mean	sd	P	*N*	mean	sd	P
Folat (nmol/L)				0.075				0.103				0.641				0.144				0.258				0.231
Quartile 1	45	14.4	5.8		43	14.1	5.9		59	13.0	5.5		45	14.0	5.4		57	12.3	8.0		35	10.3	4.8	
Quartile 2	41	12.9	5.6		39	13.8	5.2		59	12.6	5.6		45	12.5	4.3		54	11.7	5.1		34	9.5	6.2	
Quartile 3	43	12.7	4.9		41	12.9	6.3		58	11.8	5.6		44	14.3	9.5		55	10.9	6.2		36	9.4	5.7	
Quartile 4	45	12.6	5.4		40	12.1	6.5		57	13.4	6.3		41	11.3	5.1		54	11.0	6.4		31	9.0	3.4	
Ferritin (µg/L)				0.747				0.752				0.867				0.797				0.843				0.447
Quartile 1	44	30.4	12.9		42	41.4	25.5		58	28.1	22.0		45	33.9	22.0		53	31.6	24.4		35	69.8	36.8	
Quartile 2	40	33.2	20.1		36	45.7	19.2		59	28.1	23.6		43	26.6	16.5		48	25.3	15.1		32	48.9	23.1	
Quartile 3	42	35.0	19.1		41	38.1	19.0		55	27.0	21.6		42	36.6	19.1		45	40.1	37.1		33	61.3	38.0	
Quartile 4	45	30.6	16.0		39	40.6	16.7		56	29.0	25.1		40	36.1	17.9		51	28.0	21.4		29	76.2	37.2	
25(OH)D (nmol/L)				0.668				0.214				0.723				0.424				0.157				0.353
Quartile 1	43	49.8	15.3		43	57.5	17.0		59	49.9	17.3		45	53.5	13.7		56	51.5	20.6		34	46.3	17.8	
Quartile 2	38	48.7	12.3		38	54.8	13.1		59	53.9	19.0		47	55.7	16.4		54	49.3	21.1		33	49.3	15.0	
Quartile 3	43	52.6	13.2		42	55.9	11.5		59	55.0	17.8		45	52.8	15.5		56	55.6	23.5		36	49.2	14.8	
Quartile 4	42	50.4	14.7		42	53.7	17.2		55	51.7	15.2		41	52.1	17.8		56	56.4	21.4		34	41.3	15.1	
Weight status																								
BMI-sds				0.487				0.079				0.314				0.964				0.056				0.875
Quartile 1	139	0.26	1.15		116	0.51	1.18		143	0.24	1.02		119	0.22	1.10		140	0.29	0.92		100	0.32	1.17	
Quartile 2	138	0.30	1.24		120	0.60	1.13		142	0.06	1.00		118	0.17	1.08		141	0.32	0.90		101	0.41	1.15	
Quartile 3	139	0.42	1.18		122	0.38	1.18		144	0.27	0.92		119	0.13	1.06		138	0.40	0.93		102	0.17	1.04	
Quartile 4	136	0.32	0.99		121	0.31	1.17		143	0.30	0.98		119	0.24	1.16		138	0.49	0.96		101	0.38	1.16	
Dietary quality scores *																								
SHEIA15				<0.001				<0.001				<0.001				<0.001				<0.001				<0.001
Quartile 1	140	6.2	0.6		123	6.0	0.6		144	6.3	0.7		119	6.1	0.8		145	6.5	0.9		106	6.0	0.8	
Quartile 2	140	6.0	0.6		122	5.8	0.7		143	6.2	0.8		119	5.9	0.8		144	6.1	0.7		106	5.6	0.8	
Quartile 3	140	5.8	0.6		123	5.7	0.7		144	5.9	0.7		119	5.5	0.8		144	5.7	0.9		106	5.5	0.8	
Quartile 4	139	5.5	0.7		122	5.4	0.6		143	5.4	0.7		119	5.3	0.8		144	5.5	0.8		105	5.0	0.8	
RADDS				<0.001				<0.001				<0.001				<0.001				<0.001				<0.001
Quartile 1	140	6.3	1.7		123	5.8	1.9		144	6.3	1.9		119	6.1	2.1		145	6.5	2.0		106	6.2	1.809	
Quartile 2	140	6.6	1.5		122	5.9	1.5		143	6.6	1.9		119	5.9	1.9		144	6.2	1.8		106	5.6	1.783	
Quartile 3	140	6.1	1.7		123	5.7	1.8		144	6.2	1.8		119	5.5	1.9		144	5.7	1.9		106	5.5	1.858	
Quartile 4	139	5.5	1.7		122	5.2	1.7		143	5.2	1.8		119	4.7	1.8		144	5.1	1.9		105	4.3	1.491	

SHEIA15, Swedish Healthy Eating Index for Adolescents; RADDS, Riksmaten adolescents Dietary Diversity Score; BMI-sds, BMI standard deviation score; * *p*-values for linear regression, predicting nutrient level and diet scores respectively from percent energy from discretionary foods/beverages.

## Data Availability

The dataset analysed during the current study are not publicly available due to legislation protecting personal data but may be made available from the corresponding author on reasonable request if in compliance with data protection legislation.

## Supplementary Materials

The following are available online at https://www.mdpi.com/article/10.3390/nu13072450/s1, File S1: Food list with the discretionary food classification. Table S1 Contribution of discretionary foods/beverages to total intake of all foods/beverages and to total discretionary intake, by weight.
