# Supplementary material for: The Contribution of Foods and Beverages of Low Nutritional Value to the Diets of Swedish Adolescents, by Food Group, Time and Place. A Nationally Representative Study
Source: Nutrients. 2021 Jul 17;13(7):2450. doi: 10.3390/nu13072450 (PMC8308806; doi:10.3390/nu13072450)
Supplement: Supplementary file 1 [file nutrients-13-02450-s001.zip › Supplementary File S1 and Table S2/File S1 Discretionary food list.pdf]

Supplementary File 1. Foods classified as discretionary

Food item	Classed as discretionary?	Decision was based on (with respect to the Australian definition)*	Original food group	Food group as used in analysis (if different)
Carbonated soft drinks sweetened w/ synthetic sweeteners	Yes	All artificial sweeteners classed as discretionary	"Light drinks"	
Diet Cola sweetened w/ synthetic sweeteners	Yes	All artificial sweeteners classed as discretionary	"Light drinks"	
Cordial artificial sweetener RTD	Yes	All artificial sweeteners classed as discretionary	"Light drinks"	
Kelp dried	No		Algea	
Artificial sweetener	Yes	All artificial sweeteners classed as discretionary	Artificial sweetener	
Small beer or low-alcohol beer vol. % 2.3	Yes	All alcoholic drinks classed as discretionary	Beer	
Beer or pilsner vol. % 3.5	Yes	All alcoholic drinks classed as discretionary	Beer	
Strong beer vol. % 5.4	Yes	All alcoholic drinks classed as discretionary	Beer	
Non alcoholic beer	Yes	All alcoholic drinks classed as discretionary	Beer	
Raspberries	No		Berries fresh frozen	
Strawberries	No		Berries fresh frozen	
Blackberries	No		Berries fresh frozen	
Blueberries	No		Berries fresh frozen	
Berries fresh	No		Berries fresh frozen	
Currant	No		Berries fresh frozen	
Multi-vitamin beverage prepared from concentrate RTD	Yes	All sugar-sweetened beverages classed as discretionary	Beverages miscellaneous	Sugar-sweetened beverages
Water w/ vitamins and minerals added	Yes	All sugar-sweetened beverages classed as discretionary	Beverages miscellaneous	Sugar-sweetened beverages
Blood bread boiled	No		Blood products and dishes	
Black pudding fat 13% fried	Yes	Sausage with more than 5g saturated fat classed as discretionary	Blood products and dishes	
Chicken stock RTE	No		Bouillon	
Veal brawn	No	Classed as meat	Brawn	
Breakfast cereal rice sugar cocoa powder fortified Coco Pops	Yes	Breakfast cereal with more than 30g sugar/100g classed as discretionary	Breakfast cereals	
Breakfast cereal fortified Kalaspuffar	Yes	Breakfast cereal with more than 30g sugar/100g classed as discretionary	Breakfast cereals	
Breakfast cereal fortified Rice Krispies	No		Breakfast cereals	
Breakfast cereal fortified Frosties	No		Breakfast cereals	
Muesli	No		Breakfast cereals	
Granola start	No		Breakfast cereals	
Breakfast cereals rings	No		Breakfast cereals	
Breakfast cereals squares	No		Breakfast cereals	
Breakfast cereals bran flakes	No		Breakfast cereals	
Breakfast cereals corn flakes	No		Breakfast cereals	
Breakfast cereals Special K	No		Breakfast cereals	
Cereals w/ red fruit	No		Breakfast cereals	
Butter 80 % fat	Yes	Fat (butter) classed as discretionary	Butter	
Butter flavoured w/ garlic or parsley	Yes	Fat (butter) classed as discretionary	Butter	
Cocoa powder fat 20-22%	Yes	Classed as discretionary	Cacao produkts	
Sweet wheat bread cinnamon buns home-made	Yes	All cakes, biscuits classed as discretionary	Cakes, biscuits	Cakes, biscuits
Sweet wheat bread filled	Yes	All cakes, biscuits classed as discretionary	Cakes, biscuits	Cakes, biscuits
Wheat rusks	Yes	All cakes, biscuits classed as discretionary	Cakes, biscuits	Cakes, biscuits
Danish pastry filled w/ vanilla custard jam powdered sugar	Yes	All cakes, biscuits classed as discretionary	Cakes, biscuits	Cakes, biscuits
Sweet wheat bread w/ saffron	Yes	All cakes, biscuits classed as discretionary	Cakes, biscuits	Cakes, biscuits
Filled wafers biscuits	Yes	All cakes, biscuits classed as discretionary	Cakes, biscuits	Cakes, biscuits
American chocolate cookies	Yes	All cakes, biscuits classed as discretionary	Cakes, biscuits	Cakes, biscuits
Gingerbread biscuits	Yes	All cakes, biscuits classed as discretionary	Cakes, biscuits	Cakes, biscuits
Meringue	Yes	All cakes, biscuits classed as discretionary	Cakes, biscuits	Cakes, biscuits
Almond macaroons	Yes	All cakes, biscuits classed as discretionary	Cakes, biscuits	Cakes, biscuits
Layer cake filled w/ whipped cream vanilla custard raspberry jam covered w/ marzipan	Yes	All cakes, biscuits classed as discretionary	Cakes, biscuits	Cakes, biscuits
Sweet wheat bun filled w/ whipped cream and almond paste	Yes	All cakes, biscuits classed as discretionary	Cakes, biscuits	Cakes, biscuits
American muffins unspec.	Yes	All cakes, biscuits classed as discretionary	Cakes, biscuits	Cakes, biscuits
Sponge cake gingerbread cake	Yes	All cakes, biscuits classed as discretionary	Cakes, biscuits	Cakes, biscuits
Mums-mums raw meringue on wafer covered w/ chocolate	Yes	All cakes, biscuits classed as discretionary	Cakes, biscuits	Cakes, biscuits
Chocolate balls wholemeal	Yes	All cakes, biscuits classed as discretionary	Cakes, biscuits	Cakes, biscuits
Carrot cake	Yes	All cakes, biscuits classed as discretionary	Cakes, biscuits	Cakes, biscuits
Muffin queen cake	Yes	All cakes, biscuits classed as discretionary	Cakes, biscuits	Cakes, biscuits
Chocolate mud cake	Yes	All cakes, biscuits classed as discretionary	Cakes, biscuits	Cakes, biscuits
Meringue cake (Pavlova)	Yes	All cakes, biscuits classed as discretionary	Cakes, biscuits	Cakes, biscuits
Biscuit small	Yes	All cakes, biscuits classed as discretionary	Cakes, biscuits	Cakes, biscuits
Biscuit large	Yes	All cakes, biscuits classed as discretionary	Cakes, biscuits	Cakes, biscuits
Double biscuit	Yes	All cakes, biscuits classed as discretionary	Cakes, biscuits	Cakes, biscuits
Biscuit w/ chocolate coating	Yes	All cakes, biscuits classed as discretionary	Cakes, biscuits	Cakes, biscuits
Cinnamon bun crescent	Yes	All cakes, biscuits classed as discretionary	Cakes, biscuits	Cakes, biscuits

Supplementary File 1. Foods classified as discretionary

Food item	Classed as discretionary?	Decision was based on (with respect to the Australian definition)*	Original food group	Food group as used in analysis (if different)
Donut unspec.	Yes	All cakes, biscuits classed as discretionary	Cakes, biscuits	Cakes, biscuits
Mazarin bisque punch roll	Yes	All cakes, biscuits classed as discretionary	Cakes, biscuits	Cakes, biscuits
Cream cake	Yes	All cakes, biscuits classed as discretionary	Cakes, biscuits	Cakes, biscuits
Daim cake	Yes	All cakes, biscuits classed as discretionary	Cakes, biscuits	Cakes, biscuits
Brownie	Yes	All cakes, biscuits classed as discretionary	Cakes, biscuits	Cakes, biscuits
Camembert cheese white mould ripened deep-fried	Yes	Cheese that is deep-fried clased as discretionary	Cheese dish	
Halloumi cheese fried or grilled	No		Cheese dish	
Chili cheese	Yes	Cheese dish/vegetable that is deep-fried classed as discretionary	Cheese dish	
Tofu soya bean curd	No		Cheese with vegetable fat	
Tofutti (creamy smooth) original	No		Cheese with vegetable fat	
Hard cheese soy protein	No		Cheese with vegetable fat	
Chewing gum unspec.	Yes	All chewing gum classed as discretionary	Chewing gum	
Dark chocolate cocoa >70%	Yes	All sweets, chocolate classed as discretionary	Chocolate	Sweets, chocolate
Chocolate bulk confectionary	Yes	All sweets, chocolate classed as discretionary	Chocolate	Sweets, chocolate
Chocolate w/ toffee center unspec.	Yes	All sweets, chocolate classed as discretionary	Chocolate	Sweets, chocolate
Milk chocolate unspec.	Yes	All sweets, chocolate classed as discretionary	Chocolate	Sweets, chocolate
Irish Coffee	Yes	All alcoholic drinks classed as discretionary	Cider, soft drink with alcohol, drinks	
Cider vol. % 4.5	Yes	All alcoholic drinks classed as discretionary	Cider, soft drink with alcohol, drinks	
Cider vol. % 2.25	Yes	All alcoholic drinks classed as discretionary	Cider, soft drink with alcohol, drinks	
Long drink (ca 4-6 cl spirits)	Yes	All alcoholic drinks classed as discretionary	Cider, soft drink with alcohol, drinks	
Punch w/ wine	Yes	All alcoholic drinks classed as discretionary	Cider, soft drink with alcohol, drinks	
Soft drink w/ alcohol vol. % 4-5	Yes	All alcoholic drinks classed as discretionary	Cider, soft drink with alcohol, drinks	
Milkshake chocolate strawberry	No		Cocoa milkdrink milkshake smothie w youghurt	
Cocoa w/ milk fat 1,5%	No		Cocoa milkdrink milkshake smothie w youghurt	
Yoghurt drink 0.5% fat e.g. Yoggi Yalla!	No		Cocoa milkdrink milkshake smothie w youghurt	
Smoothie w/ berries yoghurt banana juice	No		Cocoa milkdrink milkshake smothie w youghurt	
Caramel frappuccino	Yes	Milk with added sugar classed as discretionary	Cocoa milkdrink milkshake smothie w youghurt	Sugar-sweetened beverages
Coffee brewed	No		Coffee infusion	
Chai latte	Yes	Coffee with added sugar classed as discretionary	Coffee Tea with milk	Sugar-sweetened beverages
Caffè latte	No		Coffee Tea with milk	
Sour cream fat 12%	Yes	All very high-fat dairy products classed as discretionary	Cream, creme fraiche	
Whipped cream fat 40%	Yes	All very high-fat dairy products classed as discretionary	Cream, creme fraiche	
Cooking cream fat 15%	Yes	All very high-fat dairy products classed as discretionary	Cream, creme fraiche	
Creme fraiche	Yes	All very high-fat dairy products classed as discretionary	Cream, creme fraiche	
Alternative cream from oats or soy beans	Yes	Classed as the non-vegetarian variant	Creme, creme fraiche	
Crisp bread thin wheat barley sugar fibre c 3% Wasa Tunnbröd	No		Crisp bread	
Wheat wholemeal rusks	No		Crisp bread	
Grissini Italian breadsticks	No		Crisp bread	
Crisp bread wholegrain	No		Crisp bread	
Crisp bread thin	No		Crisp bread	
Crisp bread white	No		Crisp bread	
Peanut doodles or rings	Yes	All crisps, savoury snacks classed as discretionary	Crisps, savoury snacks	
Corn crisps	Yes	All crisps, savoury snacks classed as discretionary	Crisps, savoury snacks	
Cheese doodles (cheese-flavored cheese puffs)	Yes	All crisps, savoury snacks classed as discretionary	Crisps, savoury snacks	
Salted sticks	Yes	All crisps, savoury snacks classed as discretionary	Crisps, savoury snacks	
Popcorn microwave fat 22%	Yes	All crisps, savoury snacks classed as discretionary	Crisps, savoury snacks	
Crisps flavoured 34% fat	Yes	All crisps, savoury snacks classed as discretionary	Crisps, savoury snacks	
Mixed savory snacks	Yes	All crisps, savoury snacks classed as discretionary	Crisps, savoury snacks	

Supplementary File 1. Foods classified as discretionary

Food item	Classed as discretionary?	Decision was based on (with respect to the Australian definition)*	Original food group	Food group as used in analysis (if different)
Crispy potato snacks	Yes	All crisps, savoury snacks classed as discretionary	Crisps, savoury snacks	
Crisps shrimp taste	Yes	All crisps, savoury snacks classed as discretionary	Crisps, savoury snacks	
Bread slices dipped in egg batter fried ('poor knights')	Yes	All desserts classed as discretionary	Desserts	Desserts, ice-cream
Glorified rice	Yes	All desserts classed as discretionary	Desserts	Desserts, ice-cream
Rice pudding	Yes	All desserts classed as discretionary	Desserts	Desserts, ice-cream
Meringues and whipped cream decorated w/ chocolate sauce 'court dessert'	Yes	All desserts classed as discretionary	Desserts	Desserts, ice-cream
Chocolate fool or chocolate pudding	Yes	All desserts classed as discretionary	Desserts	Desserts, ice-cream
Lemon mousse	Yes	All desserts classed as discretionary	Desserts	Desserts, ice-cream
Rhubarb pie	Yes	All desserts classed as discretionary	Desserts	Desserts, ice-cream
Apple pie	Yes	All desserts classed as discretionary	Desserts	Desserts, ice-cream
Cheese cake w/ crust made of digestive biscuits	Yes	All desserts classed as discretionary	Desserts	Desserts, ice-cream
Sweet pie (pie as a dessert unspec.)	Yes	All desserts classed as discretionary	Desserts	Desserts, ice-cream
Deep fried banana	Yes	All desserts classed as discretionary	Desserts	Desserts, ice-cream
Baked apples	Yes	All desserts classed as discretionary	Desserts	Desserts, ice-cream
Swedish cheesecake fat 7%	Yes	All desserts classed as discretionary	Desserts	Desserts, ice-cream
Tiramisu	Yes	All desserts classed as discretionary	Desserts	Desserts, ice-cream
Vanilla pudding Panna cotta	Yes	All desserts classed as discretionary	Desserts	Desserts, ice-cream
Baklava	Yes	All desserts classed as discretionary	Desserts	Desserts, ice-cream
Fruit berries w/ white chocolate gratinated	Yes	All desserts classed as discretionary	Desserts	Desserts, ice-cream
Meal replacment RTD e.g. Nutrilett	Yes	All dietary supplements classed as discretionary	Dietary supplements	
Meal replacement bar e.g. Nutrilett	Yes	All dietary supplements classed as discretionary	Dietary supplements	
Proteinbar	Yes	All dietary supplements classed as discretionary	Dietary supplements	
Protein supplement powder	Yes	All dietary supplements classed as discretionary	Dietary supplements	
Recovery product Gainer-Pro powder	Yes	All dietary supplements classed as discretionary	Dietary supplements	
Egg raw conventional	No		Egg	
Egg fried conventional	No		Egg	
Boiled egg conventional	No		Egg	
Farmer's omelette	No		Egg products and dishes	
French omelette w/ water	No		Egg products and dishes	
Scrambled eggs w/o flour	No		Egg products and dishes	
Margarine liquid fat 80% fortified Milda	No		Fat of mixed origin >=80%fat	
Butter and rapeseed oil liquid	Yes	Fat (oil with butter) classed as discretionary	Fat of mixed origin >=80%fat	
Small yoghurt pot size	No		Fermented milk products	
Small yoghurt pot size w/ müsli	No		Fermented milk products	
Tuna canned	No		Fish and shellfish	
Shellfish unspec.	No		Fish and shellfish	
Fish fingers breaded, fried	Yes	Fish battered/crumbed and deep-fried classed as discretionary	Fish and shellfish dishes	
Fish fritters or deep fried fish	Yes	Fish battered/crumbed and deep-fried classed as discretionary	Fish and shellfish dishes	
Shrimps deep fried restaurant	Yes	Fish battered/crumbed and deep-fried classed as discretionary	Fish and shellfish dishes	
White fish breaded and pan-fried	Yes	Fish battered/crumbed and deep-fried classed as discretionary	Fish and shellfish dishes	
Flatfish breaded pan-fried	Yes	Fish battered/crumbed and deep-fried classed as discretionary	Fish and shellfish dishes	
Cured salmon and potatoes baked in eggs	No		Fish and shellfish dishes	
Salted herring and potatoes baked in eggs	No		Fish and shellfish dishes	
Sardines canned in tomato sauce	No		Fish and shellfish dishes	
Mackerel fillets canned in tomato sauce	No		Fish and shellfish dishes	
Baltic herring fermented	No		Fish and shellfish dishes	
Herring salted coated fried home-made	No		Fish and shellfish dishes	
Herring breaded fried	No		Fish and shellfish dishes	
Fish balls w/ lobster sauce canned prepared	No		Fish and shellfish dishes	
Fish balls canned breaded fried	No		Fish and shellfish dishes	
Fish paté w/ shellfish	No		Fish and shellfish dishes	
Fish au gratin w/ dill sauce mashed potatoes	No		Fish and shellfish dishes	
Sushi diff. kinds	No		Fish and shellfish dishes	

Supplementary File 1. Foods classified as discretionary

Food item	Classed as discretionary?	Decision was based on (with respect to the Australian definition)*	Original food group	Food group as used in analysis (if different)
Fish w/ almonds bread crumbs frozen	No		Fish and shellfish dishes	
Fish au gratin w/ mashed potatoes dill sauce frozen prepared	No		Fish and shellfish dishes	
Shrimps in thick white sauce	No		Fish and shellfish dishes	
Fish paste/paté	No		Fish and shellfish dishes	
Fish and shellfish casserole	No		Fish and shellfish dishes	
Pickled herring w/ tomato sauce	No		Fish and shellfish dishes	
Pickled herring w/ sour cream	No		Fish and shellfish dishes	
Mix of egg, anchovy, red onion, creme fraiche, mayonnaise (indiviudal recipes may differ)	No		Fish and shellfish dishes	
Pickled herring	No		Fish and shellfish dishes	
Tuna and tomato sauce	No		Fish and shellfish dishes	
Pickled herring mustard sauce drained	No		Fish and shellfish dishes	
Fish gratin	No		Fish and shellfish dishes	
Croustades filled w/ mushroom stew or skagen mixture	No		Fish and shellfish dishes	
Salmon fishballs, fish burger fried	No		Fish and shellfish dishes	
Sardines canned in oil	No		Fish fresh frozen cooked	
White fish boiled	No		Fish fresh frozen cooked	
Other fish boiled	No		Fish fresh frozen cooked	
Eel baked home-made	No		Fish fried	
Salmon fried	No		Fish fried	
Baltic herring fried	No		Fish fried	
Salmon drawn smoked	No		Fish smoked	
Salmon cold smoked or dry salted	No		Fish smoked	
Fermented milk strawberry flavour 2.7% fat	No		Flavoured fermented milk	
Fermented milk skimmed strawberry flavour 0.5% fat	No		Flavoured fermented milk	
Yoghurt flavoured full fat	No		Flavoured fermented milk	
Yoghurt flavoured light	No		Flavoured fermented milk	
Rye crushed grains or flakes steamed wholemeal	No		Flour starch bran	
Wheat germs	No		Flour starch bran	
Bran	No		Flour starch bran	
Oats rolled oats	No		Flour starch bran	
Feta cheese 25% fat	No		Fresh cheese and quark	
Cream cheese fat 27% Philadelphia	No		Fresh cheese and quark	
Cream cheese extra lgt 5% fat Philadelphia	No		Fresh cheese and quark	
Mozzarella cheese fat 20%	No		Fresh cheese and quark	
Cottage cheese plain	No		Fresh cheese and quark	
Cottage cheese flavoured	No		Fresh cheese and quark	
Cottage cheese w/ fruits and nuts	No		Fresh cheese and quark	
Quark plain	No		Fresh cheese and quark	
Quark flavoured	No		Fresh cheese and quark	
Drinking quark	No		Fresh cheese and quark	
Apricots dried	No		Fruit and berries dried	
Dates dried	No		Fruit and berries dried	
Figs dried	No		Fruit and berries dried	
Prunes 32.5% moisture	No		Fruit and berries dried	
Raisins	No		Fruit and berries dried	
Fruit dried and sweetened	No		Fruit and berries dried	
Cranberries dried	No		Fruit and berries dried	
Pineapple canned in juice	No		Fruit and berries preserved	
Fruits canned	No		Fruit and berries preserved	
Energy bar chocolate nuts Start	Yes	Classed as sweets, chocolate	Fruit and nuts mixes bars	Sweets, chocolate
Sesame biscuits	Yes	Classed as sweets, chocolate	Fruit and nuts mixes bars	Sweets, chocolate
Plantains fried or deep fried	Yes	Fruit that is deep-fried classed as discretionary	Fruit fresh frozen	
Pomegranate	No		Fruit fresh frozen	
Grapefruit	No		Fruit fresh frozen	
Pineapple	No		Fruit fresh frozen	
Orange	No		Fruit fresh frozen	
Apricot	No		Fruit fresh frozen	
Banana	No		Fruit fresh frozen	
Lemon	No		Fruit fresh frozen	
Small citrus fruit clementines mandarins satsumas	No		Fruit fresh frozen	
Figs	No		Fruit fresh frozen	
Kiwi fruit green	No		Fruit fresh frozen	
Sweet cherries	No		Fruit fresh frozen	
Lime	No		Fruit fresh frozen	
Mango	No		Fruit fresh frozen	
Passion fruit	No		Fruit fresh frozen	
Peach	No		Fruit fresh frozen	

Supplementary File 1. Foods classified as discretionary

Food item	Classed as discretionary?	Decision was based on (with respect to the Australian definition)*	Original food group	Food group as used in analysis (if different)
Sharon	No		Fruit fresh frozen	
Plums	No		Fruit fresh frozen	
Pear	No		Fruit fresh frozen	
Grapes	No		Fruit fresh frozen	
Apple w/ skin	No		Fruit fresh frozen	
Fruit fresh	No		Fruit fresh frozen	
Citrus fruits	No		Fruit fresh frozen	
Fruit salad	No		Fruit fresh frozen	
Exotic fruit	No		Fruit fresh frozen	
Smoothie w/ fruits and berries	No		Fruit fresh frozen	
Melon	No		Fruit fresh frozen	
Orange juice RTD	No		Fruit juices etc	
Lemon juice fresh	No		Fruit juices etc	
Grapefruit juice fresh	No		Fruit juices etc	
Lime juice fresh	No		Fruit juices etc	
Apple juice RTD	No		Fruit juices etc	
Juice unspec.	No		Fruit juices etc	
Tropical juice RTD	No		Fruit juices etc	
Cordial reduced sugar RTD	Yes	All sugar-sweetened beverages classed as discretionary	Fruit syrup soft drink, non-alcoholic cider	Sugar-sweetened beverages
Fruit syrup RTD	Yes	All sugar-sweetened beverages classed as discretionary	Fruit syrup soft drink, non-alcoholic cider	Sugar-sweetened beverages
Soft drinks carbonated	Yes	All sugar-sweetened beverages classed as discretionary	Fruit syrup soft drink, non-alcoholic cider	Sugar-sweetened beverages
Cola drink	Yes	All sugar-sweetened beverages classed as discretionary	Fruit syrup soft drink, non-alcoholic cider	Sugar-sweetened beverages
Apple cowberry drink RTD fortified	Yes	All sugar-sweetened beverages classed as discretionary	Fruit syrup soft drink, non-alcoholic cider	Sugar-sweetened beverages
Ice tea RTD	Yes	All sugar-sweetened beverages classed as discretionary	Fruit syrup soft drink, non-alcoholic cider	Sugar-sweetened beverages
Cider non-alcoholic	Yes	All sugar-sweetened beverages classed as discretionary	Fruit syrup soft drink, non-alcoholic cider	Sugar-sweetened beverages
Fruit syrup soft drink non-carbonated	Yes	All sugar-sweetened beverages classed as discretionary	Fruit syrup soft drink, non-alcoholic cider	Sugar-sweetened beverages
Nectar w/ spices "gluhwein"	Yes	All sugar-sweetened beverages classed as discretionary	Fruit syrup soft drink, non-alcoholic cider	Sugar-sweetened beverages
Nectars RTD	Yes	All sugar-sweetened beverages classed as discretionary	Fruit syrup soft drink, non-alcoholic cider	Sugar-sweetened beverages
Cranberry drink RTD	Yes	All sugar-sweetened beverages classed as discretionary	Fruit syrup soft drink, non-alcoholic cider	Sugar-sweetened beverages
Corn flour polenta boiled	No		Grain bulgur, couscous	
Buckwheat w/ salt	No		Grain bulgur, couscous	
Quinoa red boiled w/ salt	No		Grain bulgur, couscous	
Bulgur boiled regular and wholegrain	No		Grain bulgur, couscous	
Grains boiled	No		Grain bulgur, couscous	
Couscous boiled	No		Grain bulgur, couscous	
Gruel for adults wholemeal fortified	No		Gruel	
Chicken burger Mac Donalds	No		Hamburger with bread	
Fish burger Mac Donalds	No		Hamburger with bread	
Vegetarian burger w/ bread salad sauce	No		Hamburger with bread	
Hamburger big mac whopper frisco	No		Hamburger with bread	
Hamburger small	No		Hamburger with bread	
Hamburger large big tasty super cheese n bacon	Yes	Hamburger with more than 5g saturated fat/100g classed as discretionary	Hamburger with bread	
Hamburger w/o bread	Yes	Hamburger with more than 5g saturated fat/100g classed as discretionary	Hamburger with bread	
Hard cheese fat 17%	No		Hard cheese	
Parmesan hard cheese grated 30% fat	No		Hard cheese	
Cheese fat 27%	No		Hard cheese	
Ice cream in chocolate wafers GB Sandwich	Yes	All ice-cream classed as discretionary	Ice cream	Desserts, ice-cream
Soft ice cream vanilla	Yes	All ice-cream classed as discretionary	Ice cream	Desserts, ice-cream
Ice cream cake fat 17%	Yes	All ice-cream classed as discretionary	Ice cream	Desserts, ice-cream
Sorbet	Yes	All ice-cream classed as discretionary	Ice cream	Desserts, ice-cream
Ice cream mix	Yes	All ice-cream classed as discretionary	Ice cream	Desserts, ice-cream
Ice cream in cup	Yes	All ice-cream classed as discretionary	Ice cream	Desserts, ice-cream
Ice cream bars	Yes	All ice-cream classed as discretionary	Ice cream	Desserts, ice-cream
Soft ice cream in cone	Yes	All ice-cream classed as discretionary	Ice cream	Desserts, ice-cream
Ice lolly small	Yes	All ice-cream classed as discretionary	Ice cream	Desserts, ice-cream
Ice lolly big	Yes	All ice-cream classed as discretionary	Ice cream	Desserts, ice-cream
Ice cream cone big	Yes	All ice-cream classed as discretionary	Ice cream	Desserts, ice-cream
Ice cream cone small	Yes	All ice-cream classed as discretionary	Ice cream	Desserts, ice-cream
Ice cream yoghurt ice cream	Yes	All ice-cream classed as discretionary	Ice cream	Desserts, ice-cream
Milk free Ice Cream	Yes	All ice-cream classed as discretionary	Ice cream	Desserts, ice-cream

Supplementary File 1. Foods classified as discretionary

Food item	Classed as discretionary?	Decision was based on (with respect to the Australian definition)*	Original food group	Food group as used in analysis (if different)
Ice cream cone	Yes	All ice-cream classed as discretionary	Ice cream	Desserts, ice-cream
Ice cream Ben & Jerrys	Yes	All ice-cream classed as discretionary	Ice cream	Desserts, ice-cream
Ice lolly unspec.	Yes	All ice-cream classed as discretionary	Ice cream	Desserts, ice-cream
Ice cream cake round	Yes	All ice-cream classed as discretionary	Ice cream	Desserts, ice-cream
Black currant jelly	Yes	All jam etc classed as discretionary	Jam, marmelade	
Cowberry/lingonberry jam	Yes	All jam etc classed as discretionary	Jam, marmelade	
Apple sauce	Yes	All jam etc classed as discretionary	Jam, marmelade	
Cumberland sauce	Yes	All jam etc classed as discretionary	Jam, marmelade	
Chutney	Yes	All jam etc classed as discretionary	Jam, marmelade	
Jam, strawberry, rasberry and blueberry	Yes	All jam etc classed as discretionary	Jam, marmelade	
Jam orange, apricot and rasberry	Yes	All jam etc classed as discretionary	Jam, marmelade	
Green peas	No		Leguminous plant	
Sugar pea boiled w/ salt	No		Leguminous plant	
Chickpeas canned	No		Leguminous plant	
Lentils dried boiled w/ salt	No		Leguminous plant	
Beans tinned	No		Leguminous plant	
Green beans unspec.	No		Leguminous plant	
Sprouts unspec.	No		Leguminous plant	
Pasta made of beans	No		Leguminous plant	
Liqueur sweet vol. % 24	Yes	All alcoholic drinks classed as discretionary	Liqueur	
Cucumber mayonnaise or cucumber salad fast food	Yes	All mayonnaise salads classed as discretionary	Mayonnaise salad	
Shrimp mayonnaise or shrimp salad fast food	Yes	All mayonnaise salads classed as discretionary	Mayonnaise salad	
Mimosa salad w/ fruit	Yes	All mayonnaise salads classed as discretionary	Mayonnaise salad	
Shellfish and mayonnaise mix	Yes	All mayonnaise salads classed as discretionary	Mayonnaise salad	
Tapenade	Yes	All mayonnaise salads classed as discretionary	Mayonnaise salad	
Chicken in a mayonnaise/sour cream based sauce	Yes	All mayonnaise salads classed as discretionary	Mayonnaise salad	
Beetroot salad mayonnaise	Yes	All mayonnaise salads classed as discretionary	Mayonnaise salad	
Tuna mayonnaise salad	Yes	All mayonnaise salads classed as discretionary	Mayonnaise salad	
Sailor's beef home-made	No		Meat products meat dishes	
Lobscouse corned beef and mashed potatoes	No		Meat products meat dishes	
Cabbage pudding	No		Meat products meat dishes	
Moussaka	No		Meat products meat dishes	
Chilli con carne	No		Meat products meat dishes	
Beef rolls fried	No		Meat products meat dishes	
Wiener schnitzel pork	No		Meat products meat dishes	
Reindeer thin slices casserole	No		Meat products meat dishes	
Minced meat filling for taco shells	No		Meat products meat dishes	
Country paté	No		Meat products meat dishes	
Kebab spec. w/ french fries	No		Meat products meat dishes	
Beef stew w/ beef and pork	No		Meat products meat dishes	
Minced meat gratin e.g. taco gratin	No		Meat products meat dishes	
Skewer w/ meat and vegetables	No		Meat products meat dishes	
Pork meat fried	No		Meat products meat dishes	
Steak tartare	No		Meat products meat dishes	
Meat loaf oven baked	No		Meat products meat dishes	
Meat balls pan fried	No		Meat products meat dishes	
Patty pan fried	No		Meat products meat dishes	
Minced meat rolls pan fried	No		Meat products meat dishes	
Minced meat sauce	No		Meat products meat dishes	
Döner kebab fried	No		Meat products meat dishes	
Pulled pork roasted	No		Meat products meat dishes	
Dumplings w/ pork shrimp	No		Meat products meat dishes	
Lamb curry	No		Meat products meat dishes	
Nacho plate	Yes	Cereal-based product with more than 5g saturated fat/100g classed as discretionary	Meat products meat dishes	
Nacho plate large	Yes	Cereal-based product with more than 5g saturated fat/100g classed as discretionary	Meat products meat dishes	
Skimmed milk powder fat 1%	No		Milk	
Milk low-fat	No		Milk	
Milk medium-fat	No		Milk	
Milk fat >3%	No		Milk	
Milk w/ extra protein 0.5% fat	No		Milk	
Milk flavoured extra protein 1.5% fat	No		Milk	
Condensed milk sweetened canned fat ca 9%	Yes	Milk with added sugar classed as discretionary	Milk	
Butter fat 60-80 %	Yes	Fat, high fat, classed as discretionary	Mixed origin spread	
Margarine fat 60-80%	No		Mixed origin spread	
Margarine fat 40-60%	No		Mixed origin spread	

Supplementary File 1. Foods classified as discretionary

Food item	Classed as discretionary?	Decision was based on (with respect to the Australian definition)*	Original food group	Food group as used in analysis (if different)
Chicken salad w/ dressing	No		Mixed salad	
Greek sallad	No		Mixed salad	
Tabbouleh	No		Mixed salad	
Pasta salad w/ chicken	No		Mixed salad	
Chicken salad	No		Mixed salad	
Shellfish salad shrimp salad	No		Mixed salad	
Salad w/ salmon or other fish	No		Mixed salad	
Tuna salad	No		Mixed salad	
Cheese and ham salad	No		Mixed salad	
Roast beef salad	No		Mixed salad	
Salad w/ beans chickpeas	No		Mixed salad	
Pasta salad w/ cheese and ham	No		Mixed salad	
Pasta salad w/ roast beef or other meat	No		Mixed salad	
Pasta salad w/ shrimps or other types of shellfish	No		Mixed salad	
Pasta salad w/ salmon or other fish	No		Mixed salad	
Pasta salad w/ tuna	No		Mixed salad	
Pasta salad w/ beans chickpeas	No		Mixed salad	
Salad w/ bulgur and falafel	No		Mixed salad	
Salad w/ chevré	No		Mixed salad	
Taco salad	No		Mixed salad	
Mushrooms	No		Mushroom	
Mushrooms fried	No		Mushroom	
Chanterelles fried	No		Mushroom	
Mushroom fried	No		Mushroom	
Dip for chicken nuggets	Yes	Most dressings, sauces classed as discretionary	Mustard ketchup HP sauce soja "flavouring"	
Tomato chili sauce	Yes	Most dressings, sauces classed as discretionary	Mustard ketchup HP sauce soja "flavouring"	
Tomato ketchup	Yes	Most dressings, sauces classed as discretionary	Mustard ketchup HP sauce soja "flavouring"	
HP-sauce	Yes	Most dressings, sauces classed as discretionary	Mustard ketchup HP sauce soja "flavouring"	
Mustard Swedish	Yes	Most dressings, sauces classed as discretionary	Mustard ketchup HP sauce soja "flavouring"	
Sweet chili sauce	Yes	Most dressings, sauces classed as discretionary	Mustard ketchup HP sauce soja "flavouring"	
Teryaki sauce Japanese	Yes	Most dressings, sauces classed as discretionary	Mustard ketchup HP sauce soja "flavouring"	
Soy sauce	No		Mustard ketchup HP sauce soja "flavouring"	
Fermented milk fat 3%	No		Natural fermented products	
Fermented milk skimmed fat 0.5%	No		Natural fermented products	
Yoghurt natural fat 3%	No		Natural fermented products	
Fermented milk semi-skimmed fat 1.5%	No		Natural fermented products	
Yoghurt natural skimmed fat 0.5%	No		Natural fermented products	
Greek or Turkish yoghurt 10% fat	No		Natural fermented products	
Yoghurt for cooking	No		Natural fermented products	
Peanuts dried	No		Nuts and seeds	
Peanuts roasted salted	No		Nuts and seeds	
Shredded coconut dried	No		Nuts and seeds	
Almonds dried	No		Nuts and seeds	
Mixed nuts salted	No		Nuts and seeds	
Mixed nuts	No		Nuts and seeds	
Nuts and dried fruits berries	No		Nuts and seeds	
Almond	No		Nuts and seeds	
Seeds	No		Nuts and seeds	
Peanut butter	No		Nuts and seeds	
Chili peanuts	No	Classed as nuts	Nuts and seeds	
Almonds candied	Yes	Classed as sweets, chocolate	Nuts and seeds	Sweets, chocolate
Liver patties fried home-made	No		Offal products and dishes	
Liver pan fried	No		Offal products and dishes	
Liver casserole unspec.	No		Offal products and dishes	
Hash made w/ eg barley offal heated fried	Yes	Classed as sausage with more than 5g saturated fat/100g discretionary	Offal products and dishes	
Offals	No		Offals	
Coconut butter	Yes		Other fats (lard, tallow, coconut oil)	
Pancakes thin home-made	No		Panncake,	
Pancake thin filled unspec.	No		Panncake,	
Pancake thick pancake w/ fried pork	No		Panncake,	
Waffles plain or egg waffles	No		Panncake,	
Pasta boiled w/ salt	No		pasta	
Pasta whole grain >50% boiled w/ salt	No		pasta	

Supplementary File 1. Foods classified as discretionary

Food item	Classed as discretionary?	Decision was based on (with respect to the Australian definition)*	Original food group	Food group as used in analysis (if different)
Noodles boiled	No		pasta	
Stir fry vegetables w/ chicken noodles	No		Pasta dishes	
Stir fry vegetables w/ noodles	No		Pasta dishes	
Pasta macaroni in thick white sauce	No		Pasta dishes	
Pasta ravioli w/ sauce frozen or canned	No		Pasta dishes	
Tortellini w/ meat filling	No		Pasta dishes	
Lasagne veg.	No		Pasta dishes	
Lasagna w/ minced beef or beef and pork mince	No		Pasta dishes	
Pasta au gratin w/ cheese sauce and ham	No		Pasta dishes	
Stir-fried noodles w/ meat, chicken or sausage	No		Pasta dishes	
Pasta gratin	No		Pasta dishes	
Pasta carbonara	No		Pasta dishes	
Ciabatta sandwich w/ mozzarella cheese sundried tomatoes and green salad	No		Pies, pizza, toasted sandwiches	
Spring roll frozen heated	No		Pies, pizza, toasted sandwiches	
Pirogue filled w/ minced meat	No		Pies, pizza, toasted sandwiches	
Pitta bread w/ falafel green salad sesame and pepperoni	No		Pies, pizza, toasted sandwiches	
Savory muffin coarse wholemeal flour w/ goat's or sheep's cheese broccoli spinach and sundried tomatoes	No		Pies, pizza, toasted sandwiches	
Savory sandwich layer-cake filled w/ liver paté cottage cheese ham	No		Pies, pizza, toasted sandwiches	
Döner kebab	No		Pies, pizza, toasted sandwiches	
Bagel w/ spread	No		Pies, pizza, toasted sandwiches	
French roll w/ spread	No		Pies, pizza, toasted sandwiches	
Foccacia w/ spread	No		Pies, pizza, toasted sandwiches	
Wrap w/ spread	No		Pies, pizza, toasted sandwiches	
Sandwich w/ spread	No		Pies, pizza, toasted sandwiches	
Baguette sub w/ spread	No		Pies, pizza, toasted sandwiches	
Subway sandwich	No		Pies, pizza, toasted sandwiches	
Enchiladas	No		Pies, pizza, toasted sandwiches	
Wheat crisp bread sandwich average diff. fillings	Yes	Cereal-based product with more than 5g saturated fat/100g classed as discretionary	Pies, pizza, toasted sandwiches	
Vegetarian pirogue	Yes	Cereal-based product with more than 5g saturated fat/100g classed as discretionary	Pies, pizza, toasted sandwiches	
Toast w/ spread	Yes	Cereal-based product with more than 5g saturated fat/100g classed as discretionary	Pies, pizza, toasted sandwiches	
Grilled sandwich w/ spread	Yes	Cereal-based product with more than 5g saturated fat/100g classed as discretionary	Pies, pizza, toasted sandwiches	
Meat pie	Yes	Pies with more than 5g saturated fat/100g classed as discretionary	Pies, pizza, toasted sandwiches	
Chicken pie	Yes	Pies with more than 5g saturated fat/100g classed as discretionary	Pies, pizza, toasted sandwiches	
Shellfish pie frozen heated	Yes	Pies with more than 5g saturated fat/100g classed as discretionary	Pies, pizza, toasted sandwiches	
Cheese and ham pie	Yes	Pies with more than 5g saturated fat/100g classed as discretionary	Pies, pizza, toasted sandwiches	
Cheese pie	Yes	Pies with more than 5g saturated fat/100g classed as discretionary	Pies, pizza, toasted sandwiches	
Vegetable pie	Yes	Pies with more than 5g saturated fat/100g classed as discretionary	Pies, pizza, toasted sandwiches	
Vegetarian pie w/ quorn	Yes	Pies with more than 5g saturated fat/100g classed as discretionary	Pies, pizza, toasted sandwiches	
Puff pastry or filo pastry baked cheese	Yes	Pies with more than 5g saturated fat/100g classed as discretionary	Pies, pizza, toasted sandwiches	
Pizza homemade	No		Pies, pizza, toasted sandwiches	
Pizza frozen or chilled	No		Pies, pizza, toasted sandwiches	
Pizza pan pizza smoked pork frozen heated	Yes	Pizza with more than 5g saturated fat/100g classed as discretionary	Pies, pizza, toasted sandwiches	
Pizza unspec.	Yes	Pizza with more than 5g saturated fat/100g classed as discretionary	Pies, pizza, toasted sandwiches	
Mini pizzas w/ ham cheese tomatoes and/or other fillings	Yes	Pizza with more than 5g saturated fat/100g classed as discretionary	Pies, pizza, toasted sandwiches	
Rice porridge	No		Porridge	
Oatmeal porridge wholemeal	No		Porridge	
Semolina porridge	No		Porridge	
Porridge multigrain	No		Porridge	
Rice porridge e.g. Risifrutti Manna	Yes	Classed as dessert	Porridge	Desserts, ice-cream
Rice pudding w/ fruit sauce e.g. Risifrutti original	Yes	Classed as dessert	Porridge	Desserts, ice-cream

Supplementary File 1. Foods classified as discretionary

Food item	Classed as discretionary?	Decision was based on (with respect to the Australian definition)*	Original food group	Food group as used in analysis (if different)
Old potato boiled w/ salt	No		Potatoes	
Potato w/ skins unsalted baked	No		Potatoes	
Potato wedges w/ skin oven-baked in rapeseed oil	No		Potatoes	
Pressed potatoes	No		Potatoes	
New potatoe boiled w/ salt	No		Potatoes	
Potato fried	No		Potatoes	
Potato dumplings stuffed w/ chopped pork	No		Potatoes products and dishes	
Potato salad w/ sour cream mayonnaise	No		Potatoes products and dishes	
Potato salad w/ vinaigrette	No		Potatoes products and dishes	
Potato pancakes home-made	No		Potatoes products and dishes	
Potatoes creamed	No		Potatoes products and dishes	
Potato patties	No		Potatoes products and dishes	
Mashed potatoes	No		Potatoes products and dishes	
Mashed potatoes instant powder fortified prepared	No		Potatoes products and dishes	
Potatoes au gratin w/ cream and cheese frozen heated	No		Potatoes products and dishes	
Potato gnocchi	No		Potatoes products and dishes	
Swedish chicken hash	No		Potatoes products and dishes	
Swedish hash in cast iron frying pan home-made	Yes	Potato dish with more than 5g saturated fat/100g classed as discretionary	Potatoes products and dishes	
Potatoes and vegetables fried	Yes	Potato dish with more than 5g saturated fat/100g classed as discretionary	Potatoes products and dishes	
Swiss potato cake rösti frozen heated	Yes	Potato dish with more than 5g saturated fat/100g classed as discretionary	Potatoes products and dishes	
Potato croquettes frozen heated	Yes	Potato dish with more than 5g saturated fat/100g classed as discretionary	Potatoes products and dishes	
Potatoes ovenbaked w/ butter, salt and breadcrumbs	Yes	Potato dish with more than 5g saturated fat/100g classed as discretionary	Potatoes products and dishes	
French fries	Yes	Potato dish with more than 5g saturated fat/100g classed as discretionary	Potatoes products and dishes	
Swedish potato gratin w/ pickled sprats coffee cream and milk Jansson's temptation	Yes	Potato dish with more than 5g saturated fat/100g classed as discretionary	Potatoes products and dishes	
Potatoes au gratin w/ ham or other meat	Yes	Potato dish with more than 5g saturated fat/100g classed as discretionary	Potatoes products and dishes	
Duck roasted	No		Poultry	
Chicken hen boiled grilled fried	No		Poultry	
Smoked turkey	No		Poultry	
Game bird boiled	No		Poultry	
Fajitas chicken	No		Poultry products and dishes	
Quesadillas	No		Poultry products and dishes	
Chicken meat balls fat 12% fried frozen	No		Poultry products and dishes	
Chicken au gratin w/ bananas bacon and peanuts (Flygande Jacob)	No		Poultry products and dishes	
Chicken casserole w/ cream/crème fraiche	No		Poultry products and dishes	
Chicken stew w/ tomato	No		Poultry products and dishes	
Pulled chicken roasted	No		Poultry products and dishes	
Chicken curry stew	No		Poultry products and dishes	
Chicken deep-fried Chicken McNuggets	Yes	Poultry that is battered and deep-fried classed as discretionary	Poultry products and dishes	
Processed cheese	No		Processed cheese	
Liver paste or paté slicable fat c 26%	No		Processed meat	
Horse meat smoked	Yes	All processed meat classed as discretionary	Processed meat	
Beef meat cured boiled	Yes	All processed meat classed as discretionary	Processed meat	
Reindeer meat dried	Yes	All processed meat classed as discretionary	Processed meat	
Bacon pork fried	Yes	All processed meat classed as discretionary	Processed meat	
Italian prosciutto cured air-dried ham pork	Yes	All processed meat classed as discretionary	Processed meat	
Smoked ham pork fat 1-3% sliced	Yes	All processed meat classed as discretionary	Processed meat	
Cured ham Christmas ham pork fat 4.5% boiled	Yes	All processed meat classed as discretionary	Processed meat	
Pork loin smoked fried or heated	Yes	All processed meat classed as discretionary	Processed meat	
Cured pork belly fried	Yes	All processed meat classed as discretionary	Processed meat	
Smoked meat	Yes	All processed meat classed as discretionary	Processed meat	
Boiled ham	Yes	All processed meat classed as discretionary	Processed meat	
Beef cube roll fried	No		Red meat fresh frozen cooked	
Spare ribs pork barbecued	No		Red meat fresh frozen cooked	
Top round steak fried (thin slices)	No		Red meat fresh frozen cooked	
Roasted steak	No		Red meat fresh frozen cooked	
Meat thin slices pan-fried	No		Red meat fresh frozen cooked	
Lamb racks	No		Red meat fresh frozen cooked	

Supplementary File 1. Foods classified as discretionary

Food item	Classed as discretionary?	Decision was based on (with respect to the Australian definition)*	Original food group	Food group as used in analysis (if different)
Meat unspec. boiled	No		Red meat fresh frozen cooked	
Beef carpaccio	No		Red meat fresh frozen cooked	
Minced meat unspec. Fried	No		Red meat fresh frozen cooked	
Chops loin collar chaps pan fried	No		Red meat fresh frozen cooked	
Loin pan fried	No		Red meat fresh frozen cooked	
Steak pan fried	No		Red meat fresh frozen cooked	
Meat grilled	No		Red meat fresh frozen cooked	
Pulled beef	No		Red meat fresh frozen cooked	
Rice cake	No		Rice cakes	
Paella	No		Rice dishes	
Rice persian	No		Rice dishes	
Fried rice w/ meat chicken or sausage	No		Rice dishes	
Rice w/ onion pilaff rice	No		Rice dishes	
Italian risotto w/ parmesan cheese	No		Rice dishes	
Wild rice boiled	No		Rice rice noodles	
Rice diff. kinds boiled w/ salt	No		Rice rice noodles	
Cellophane noodles boiled w/ salt	No		Rice rice noodles	
Long-grain brown rice boiled	No		Rice rice noodles	
Fish spawn	No	Classed as fish	Roe, roe spread	
Fish spawn paste spread smoked	No	Classed as fish	Roe, roe spread	
Beetroot pan-fried or stir-fried	No		Root vegetables	
Vegetables and root vegetables baked	No		Root vegetables	
Ginger pickles	No		Root vegetables	
Mixed vegetables carrot parsnip leek celeriac boiled	No		Root vegetables	
Sweet potato fried	No		Root vegetables	
Swede fried or woked	No		Root vegetables	
Wasabi	No		Root vegetables	
Swede	No		Root vegetables	
Carrot	No		Root vegetables	
Horseradish	No		Root vegetables	
Radish	No		Root vegetables	
Beetroot	No		Root vegetables	
Beetroots pickled drained	No		Root vegetables	
Carrot pan-fried or stir-fried	No		Root vegetables	
Celeriac pan-fried or stir-fried	No		Root vegetables	
Plain wafers	No		Salty biscuits	
Cracker small	Yes	Savoury biscuit with more than 1800 kJ/100g	Salty biscuits	
Cracker semi-sweet biscuit big	Yes	Savoury biscuit with more than 1800 kJ/100g	Salty biscuits	
Mushrooms in white sauce	No		Sauces, dressings	
White sauce béchamel	No		Sauces, dressings	
Blue cheese sauce made w/ coffee cream and whipping cream	No		Sauces, dressings	
Tomato sauce	No		Sauces, dressings	
Carbonara	No		Sauces, dressings	
Cheese and ham sauce	No		Sauces, dressings	
Cheese sauce ready-made sauce fat 8%	No		Sauces, dressings	
Shellfish sauce	No		Sauces, dressings	
Mayonnaise 90% fat	Yes	Most dressings, sauces classed as discretionary	Sauces, dressings	
Sauce (mustard based) for raw spiced salmon	Yes	Most dressings, sauces classed as discretionary	Sauces, dressings	
Rémoulade sauce	Yes	Most dressings, sauces classed as discretionary	Sauces, dressings	
Vinaigrette w/ water 45% fat	Yes	Most dressings, sauces classed as discretionary	Sauces, dressings	
Béarnaise sauce	Yes	Most dressings, sauces classed as discretionary	Sauces, dressings	
Rhode Island sauce	Yes	Most dressings, sauces classed as discretionary	Sauces, dressings	
Egg sauce w/ parsley	Yes	Most dressings, sauces classed as discretionary	Sauces, dressings	
Pesto sauce w/ basil pine nuts and cashew nuts	Yes	Most dressings, sauces classed as discretionary	Sauces, dressings	
Peanut sauce	Yes	Most dressings, sauces classed as discretionary	Sauces, dressings	
Curry sauce RTE refrigerated/frozen	Yes	Most dressings, sauces classed as discretionary	Sauces, dressings	
Hamburger dressing	Yes	Most dressings, sauces classed as discretionary	Sauces, dressings	
Barbeque sauce	Yes	Most dressings, sauces classed as discretionary	Sauces, dressings	
Whipped cream w/ horseraddish	Yes	Most dressings, sauces classed as discretionary	Sauces, dressings	

Supplementary File 1. Foods classified as discretionary

Food item	Classed as discretionary?	Decision was based on (with respect to the Australian definition)*	Original food group	Food group as used in analysis (if different)
Ceasar dressing	Yes	Most dressings, sauces classed as discretionary	Sauces, dressings	
Aioli mayonnaise	Yes	Most dressings, sauces classed as discretionary	Sauces, dressings	
Dip for french fries bearnaise	Yes	Most dressings, sauces classed as discretionary	Sauces, dressings	
Pizza topping sauce	Yes	Most dressings, sauces classed as discretionary	Sauces, dressings	
Garlic sauce	Yes	Most dressings, sauces classed as discretionary	Sauces, dressings	
Brown sauce w/ milk	No		Sauces, dressings	
Thin gravy	No		Sauces, dressings	
Tomato sauce Mexican style	No	Classed as vegetable**	Sauces, dressings	
Cold sauce school coleslaw	No	Classed as vegetable**	Sauces, dressings	
Tzatziki	No	Classed as yoghurt**	Sauces, dressings	
Yoghurt sauce	No	Classed as yoghurt**	Sauces, dressings	
Sausage Stroganoff w/ cooking cream	Yes	Sausage with more than 5g saturated fat classed as discretionary	Sausage dishes	
Sausage casserole w/ sausage chorizo vegetables root vegetables	Yes	Sausage with more than 5g saturated fat classed as discretionary	Sausage dishes	
Hot dog frankfurter made of chicken turkey or ostrich boiled	No		Sausages	
Sausage meat 48% boiled e.g. fläskkorv	Yes	Sausage with more than 5g saturated fat classed as discretionary	Sausages	
Salami sausage fat 33% smoked	Yes	Sausage with more than 5g saturated fat classed as discretionary	Sausages	
Sausage meat 73% fried e.g. Chorizo	Yes	Sausage with more than 5g saturated fat classed as discretionary	Sausages	
Salami imported fat 45%	Yes	Sausage with more than 5g saturated fat classed as discretionary	Sausages	
Sausage ('Falu' sausage) pan-fried	Yes	Sausage with more than 5g saturated fat classed as discretionary	Sausages	
Sausage fermented meat fried 59% e.g. Småländska isterband	Yes	Sausage with more than 5g saturated fat classed as discretionary	Sausages	
Sausage hot dog fried	Yes	Sausage with more than 5g saturated fat classed as discretionary	Sausages	
Sausage for sandwich unspec.	Yes	Sausage with more than 5g saturated fat classed as discretionary	Sausages	
Miniature frankfurter('Prince' sausage) pan-fried	Yes	Sausage with more than 5g saturated fat classed as discretionary	Sausages	
Shrimps raw	No		Shellfish squid fresh frozen cooked	
White bread wheat scones	Yes	Classed as cakes, biscuits	Soft bread	Cakes, biscuits
Croissant	Yes	Classed as cakes, biscuits	Soft bread	Cakes, biscuits
White bread e.g. pita fibre c 3.5%	No		Soft bread	
Hot dog bun	No		Soft bread	
White bread e.g. Italian unsweetened fibre c 2.5%	No		Soft bread	
Baguette white bread wheat	No		Soft bread	
White bread tortilla fibre 4.6%	No		Soft bread	
Bread soft thin w/ milk fibre c 4%	No		Soft bread	
Bagel	No		Soft bread	
Croutons, plain	No		Soft bread	
White bread fibre c 5% e.g. Jättefranska	No		Soft bread	
Hamburger bread	No		Soft bread	
Indian naan bread	No		Soft bread	
Bread deep fried e.g langos	No		Soft bread	
Semi whole grain bread	No		Soft bread	
Tea biscuit	No		Soft bread	
Whole grain bread	No		Soft bread	
Camembert cheese white mould ripened low fat fat 11%	No		Soft cheese	
Cheese chèvre french goat milk white mold ripened fat 25%	No		Soft cheese	
Cheese for dessert	No		Soft cheese	
Vegetable soup w/ cream	No		Soup	
Instant noodles soup diff. flavours	No		Soup	
Pea soup w/ pork canned prepared w/ water	No		Soup	
Lentil soup	No		Soup	
Pea soup veg.	No		Soup	
Beef soup	No		Soup	
Chicken soup clear	No		Soup	
Instant chicken soup cream of cooked w/ water e.g. Varma koppen	No		Soup	
Fish soup	No		Soup	
Miso soup w/ tofu	No		Soup	

Supplementary File 1. Foods classified as discretionary

Food item	Classed as discretionary?	Decision was based on (with respect to the Australian definition)*	Original food group	Food group as used in analysis (if different)
Vegetable paste Tartex	No	Classed as the non-vegetarian variant	Soy and wheat protein quorn products and dishes	
Soya sausage	No	Classed as the non-vegetarian variant	Soy and wheat protein quorn products and dishes	
Vegetarian lasagne	No	Classed as the non-vegetarian variant	Soy and wheat protein quorn products and dishes	
Vegetarian moussaka	No	Classed as the non-vegetarian variant	Soy and wheat protein quorn products and dishes	
Vegetarian schnitzel	No	Classed as the non-vegetarian variant	Soy and wheat protein quorn products and dishes	
Vegetarian swedish hash	No	Classed as the non-vegetarian variant	Soy and wheat protein quorn products and dishes	
Vegetarian filet	No	Classed as the non-vegetarian variant	Soy and wheat protein quorn products and dishes	
Vegetarian nuggets	Yes	Classed as the non-vegetarian variant	Soy and wheat protein quorn products and dishes	
Vegetarian balls	No	Classed as the non-vegetarian variant	Soy and wheat protein quorn products and dishes	
Vegetarian bolognese sauce	No	Classed as the non-vegetarian variant	Soy and wheat protein quorn products and dishes	
Vegetarian stew	No	Classed as the non-vegetarian variant	Soy and wheat protein quorn products and dishes	
Vegetarian beef	No	Classed as the non-vegetarian variant	Soy and wheat protein quorn products and dishes	
Vegetarian minced quorn soy	No	Classed as the non-vegetarian variant	Soy and wheat protein quorn products and dishes	
Cinnamon	No		Spices	
Vodka or snaps vol. % 40	Yes	All alcoholic drinks classed as discretionary	Spirits	
Energy drink e.g. Red Bull fortified	Yes	All sugar-sweetened beverages classed as discretionary	Sports drinks, energy drinks	Sugar-sweetened beverages
Sugar	Yes	All sugar, honey etc classed as discretionary	Sugar, syrup, honey	
Golden syrup	Yes	All sugar, honey etc classed as discretionary	Sugar, syrup, honey	
Honey	Yes	All sugar, honey etc classed as discretionary	Sugar, syrup, honey	
Glucose	Yes	All sugar, honey etc classed as discretionary	Sugar, syrup, honey	
Stewed fruit and berries	Yes	All sweet soups, sauces classed as discretionary	Sweet soups, sauces	Desserts, ice-cream
Fruit syrup sauce thickened w/ potato starch	Yes	All sweet soups, sauces classed as discretionary	Sweet soups, sauces	Desserts, ice-cream
Fruit drink blueberry	Yes	All sweet soups, sauces classed as discretionary	Sweet soups, sauces	Desserts, ice-cream
Fruit soup	Yes	All sweet soups, sauces classed as discretionary	Sweet soups, sauces	Desserts, ice-cream
Rosehip soup RTE pasteurised or powder prepared fortified	Yes	All sweet soups, sauces classed as discretionary	Sweet soups, sauces	Desserts, ice-cream
Soup fruit berries sweetener unspec.	Yes	All sweet soups, sauces classed as discretionary	Sweet soups, sauces	Desserts, ice-cream
Chocolate sauce	Yes	All sweet soups, sauces classed as discretionary	Sweet soups, sauces	Desserts, ice-cream
Butterscotch sauce	Yes	All sweet soups, sauces classed as discretionary	Sweet soups, sauces	Desserts, ice-cream
Custard	Yes	All sweet soups, sauces classed as discretionary	Sweet soups, sauces	Desserts, ice-cream
Berry sauce	Yes	All sweet soups, sauces classed as discretionary	Sweet soups, sauces	Desserts, ice-cream
Lollipop	Yes	All sweets, chocolate classed as discretionary	Sweets excluding chocolate	Sweets, chocolate
Candy straps cane	Yes	All sweets, chocolate classed as discretionary	Sweets excluding chocolate	Sweets, chocolate
Bulk confectionary in small box	Yes	All sweets, chocolate classed as discretionary	Sweets excluding chocolate	Sweets, chocolate
Chocolate hazelnut spread Nutella	Yes	All sweets, chocolate classed as discretionary	Sweets miscellaneous	Sweets, chocolate
Chocolate bar filled wafers covered w/ milk chocolate	Yes	All sweets, chocolate classed as discretionary	Sweets miscellaneous	Sweets, chocolate
Cereal bar enriched e.g. Special K Bar Chocolate	Yes	All sweets, chocolate classed as discretionary	Sweets miscellaneous	Sweets, chocolate
Bulk confectionary	Yes	All sweets, chocolate classed as discretionary	Sweets miscellaneous	Sweets, chocolate
Nature candy	Yes	All sweets, chocolate classed as discretionary	Sweets miscellaneous	Sweets, chocolate
Pastilles sugar free	Yes	All sugar-free sweets classed as discretionary	Sweets sugar-free	
Taco shells	No		Taco shell	
Tea brewed	No		Tea infusion	
Carrot juice	No		Vegetable juice	

Supplementary File 1. Foods classified as discretionary

Food item	Classed as discretionary?	Decision was based on (with respect to the Australian definition)*	Original food group	Food group as used in analysis (if different)
Tomato juice canned RTD	No		Vegetable juice	
Vegetable juice canned or pasteurised RTD	No		Vegetable juice	
Stir-fry vegetable mix Classic woked in rapeseed oil	No		Vegetable mixes w root vegetables and or leguminous plants	
Peas and carrots boiled	No		Vegetable mixes w root vegetables and or leguminous plants	
Mixed vegetables and root crops fried	No		Vegetable mixes w root vegetables and or leguminous plants	
Olive oil	No		Vegetable oils	
Rapeseed oil	No		Vegetable oils	
Oat milk soy milk	No	Classed as the non-vegetarian variant	Vegetable products and milk replacement	
Oatgurt Soygurt milkfree yoghurt	No	Classed as the non-vegetarian variant	Vegetable products and milk replacement	
Avocado	No		Vegetables excluding potatoes	
Celery	No		Vegetables excluding potatoes	
Cauliflower	No		Vegetables excluding potatoes	
Fennel	No		Vegetables excluding potatoes	
Kale	No		Vegetables excluding potatoes	
Cucumber	No		Vegetables excluding potatoes	
Iceberg lettuce	No		Vegetables excluding potatoes	
Onions yellow	No		Vegetables excluding potatoes	
Leek	No		Vegetables excluding potatoes	
Red cabbage	No		Vegetables excluding potatoes	
Chinese cabbage	No		Vegetables excluding potatoes	
Summer squash	No		Vegetables excluding potatoes	
Tomato	No		Vegetables excluding potatoes	
Cabbage	No		Vegetables excluding potatoes	
Lettuce mixed cucumber tomato w/o dressing	No		Vegetables excluding potatoes	
Sweet peppers green yellow red	No		Vegetables excluding potatoes	
Corn kernels canned drained	No		Vegetables excluding potatoes	
Olives green marinated drained	No		Vegetables excluding potatoes	
Sauerkraut canned whole contents	No		Vegetables excluding potatoes	
Chopped tomatoes canned whole contents	No		Vegetables excluding potatoes	
Salad lettuce mix w/ cucumber tomato sweet pepper w/o dressing	No		Vegetables excluding potatoes	
Onion fried	No		Vegetables excluding potatoes	
Spinach fried w/o browning	No		Vegetables excluding potatoes	
Aubergine fried	No		Vegetables excluding potatoes	
Cucumber pickled	No		Vegetables excluding potatoes	
Globe artichoke boiled	No		Vegetables excluding potatoes	
Corn cobs boiled	No		Vegetables excluding potatoes	
Cauliflower pan-fried or stir-fried	No		Vegetables excluding potatoes	
Fennel pan-fried or stir-fried	No		Vegetables excluding potatoes	
Sweet/bell pepper grilled, pan-fried or stir-fried	No		Vegetables excluding potatoes	
Pumpkin or marrow pan-fried or stir-fried	No		Vegetables excluding potatoes	
Leek pan-fried or stir-fried	No		Vegetables excluding potatoes	
White cabbage pan-fried or stir-fried	No		Vegetables excluding potatoes	
Salad, various kinds	No		Vegetables excluding potatoes	
Sprouts	No		Vegetables excluding potatoes	
Lettuce mixed cucumber tomato corn w/ out dressing	No		Vegetables excluding potatoes	
Vegetables brawned	No		Vegetables excluding potatoes	
Asparagus fried or woked	No		Vegetables excluding potatoes	
Corn fried	No		Vegetables excluding potatoes	
Brussels sprouts fried or woked	No		Vegetables excluding potatoes	
Tomato fried	No		Vegetables excluding potatoes	
Broccoli boiled	No		Vegetables excluding potatoes	
Spinach Mangold	No		Vegetables excluding potatoes	
Herbs	No		Vegetables excluding potatoes	
Guacamole	No		Vegetables excluding potatoes	
Coconut water natural or flavoured	No		Vegetables excluding potatoes	
Vegetable salad w/ carrot cabbage etc	No		Vegetables leguminous plant root vegetables dishes and products	
Vegetables unspec. deep fried	No		Vegetables leguminous plant root vegetables dishes and products	

Supplementary File 1. Foods classified as discretionary

Food item	Classed as discretionary?	Decision was based on (with respect to the Australian definition)*	Original food group	Food group as used in analysis (if different)
Vegetable paté	No		Vegetables leguminous plant root vegetables dishes and products	
Vegetable gratin	No		Vegetables leguminous plant root vegetables dishes and products	
Root vegetable gratin	No		Vegetables leguminous plant root vegetables dishes and products	
Burrito	No		Vegetables leguminous plant root vegetables dishes and products	
Mashed swedes and potatoes home-made	No		Vegetables leguminous plant root vegetables dishes and products	
Sundried tomatoes marinated in oil	No		Vegetables leguminous plant root vegetables dishes and products	
Ajvar relish vegetable paste w/ sweet peppers and aubergine	No		Vegetables leguminous plant root vegetables dishes and products	
Vegetable patties fried	No		Vegetables leguminous plant root vegetables dishes and products	
Cabbage red and apples fried boiled	No		Vegetables leguminous plant root vegetables dishes and products	
Mushrooms in thick white sauce	No		Vegetables leguminous plant root vegetables dishes and products	
Cabbage salad Swedish pizza salad	No		Vegetables leguminous plant root vegetables dishes and products	
Vegetables in thick white sauce	No		Vegetables leguminous plant root vegetables dishes and products	
Vegetable casserole unspec.	No		Vegetables leguminous plant root vegetables dishes and products	
Cucumber mix pickled	No		Vegetables leguminous plant root vegetables dishes and products	
Vegetables mixed fermented	No		Vegetables leguminous plant root vegetables dishes and products	
Wine leaves stuffed w/ rice canned	No		Vegetables leguminous plant root vegetables dishes and products	
White beans baked in tomato sauce canned	No		Vegetables leguminous plant root vegetables dishes and products	
Falafel chickpea-croquettes frozen	No		Vegetables leguminous plant root vegetables dishes and products	
Ratatouille	No		Vegetables leguminous plant root vegetables dishes and products	
Vegetarian casserole w/ beans and lentils	No		Vegetables leguminous plant root vegetables dishes and products	
Marinated beans	No		Vegetables leguminous plant root vegetables dishes and products	
Marinated lentils	No		Vegetables leguminous plant root vegetables dishes and products	
Marinated chickpeas	No		Vegetables leguminous plant root vegetables dishes and products	
Marinated garlic	No		Vegetables leguminous plant root vegetables dishes and products	
Aubergine mix	No		Vegetables leguminous plant root vegetables dishes and products	
Hummus dip/spread made from chickpeas	No		Vegetables leguminous plant root vegetables dishes and products	

Supplementary File 1. Foods classified as discretionary

Food item	Classed as discretionary?	Decision was based on (with respect to the Australian definition)*	Original food group	Food group as used in analysis (if different)
Swedish brown beans roll	No		Vegetables leguminous plant root vegetables dishes and products	
Roasted onion	Yes	Vegetable that is deep-fried classed as discretionary	Vegetables leguminous plant root vegetables dishes and products	
Onion rings deep fried	Yes	Vegetable that is deep-fried classed as discretionary	Vegetables leguminous plant root vegetables dishes and products	
Mineral water	No		Water, mineral water	
Tap water	No		Water, mineral water	
Spring water packed	No		Water, mineral water	
Whey cheese fat 30% fortified	No	Classed as cheese	Whey products	
Whey spread fat 5% fortified	No	Classed as cheese	Whey products	
White wine vol. % 1	Yes	All alcoholic drinks classed as discretionary	Wine	
Red wine vol. % 14	Yes	All alcoholic drinks classed as discretionary	Wine	
White wine vol. % 12	Yes	All alcoholic drinks classed as discretionary	Wine	
Wine mulled vol. % 10	Yes	All alcoholic drinks classed as discretionary	Wine	
Sherry medium dry vol. % 17	Yes	All alcoholic drinks classed as discretionary	Wine	
Fortified wine mulled vol. % 16	Yes	All alcoholic drinks classed as discretionary	Wine	
Mulled wine (non-alcoholic)	Yes	All alcoholic drinks classed as discretionary	Wine	
Sparkling wine champagne	Yes	All alcoholic drinks classed as discretionary	Wine	

* Either the entire food group was considered a discretionary food group, the food met a specified criteria as described, or the food did not have an equivalent in the Australian system and our classification is provided.

** Based on the composition of the foods in the Swedish database, we deviated from the Australian classification
