# Supplementary material for: The Contribution of Foods and Beverages of Low Nutritional Value to the Diets of Swedish Adolescents, by Food Group, Time and Place. A Nationally Representative Study
Source: Nutrients. 2021 Jul 17;13(7):2450. doi: 10.3390/nu13072450 (PMC8308806; doi:10.3390/nu13072450)
Supplement: Supplementary file 1 [file nutrients-13-02450-s001.zip › Supplementary File S1 and Table S2/Table S2.pdf]

Table S2 Contribution of meal types to consumption of total energy, the proportion of each meal (by energy) that is from discretionary foods/beverages, and the top three discretionary foods/beverages contributing most to total energy at each meal type, by age and gender

School grade: 5th						8th						11th					
Girls (N=599)			Boys (N=490)			Girls (N=574)			Boys (N=476)			Girls (N=577)			Boys (N=423)		
	% of total E	% of which is disc. E		% of total E	% of which is disc. E		% of total E	% of which is disc. E		% of total E	% of which is disc. E		% of total E	% of which is disc. E		% of total E	% of which is disc. E
Breakfast	20.1	19		20.1	19		18.6	19		19.0	20		18.0	20		16.7	20
Mixed fat spread*	1.0		Pies, pizza, toasted sandwiches*	0.9		Mixed fat spread*	0.9		Pies, pizza, toasted sandwiches*	1.1		Mixed fat spread*	0.9		Pies, pizza, toasted sandwiches*	0.8	
Pies, pizza, toasted sandwiches*	0.9		Mixed fat spread*	0.8		Pies, pizza, toasted sandwiches*	0.9		Mixed fat spread*	0.8		Pies, pizza, toasted sandwiches*	0.6		Mixed fat spread*	0.6	
Cakes, biscuits	0.3		Jam, marmelade	0.4		Cakes, biscuits	0.2		Cakes, biscuits	0.3		Cakes, biscuits	0.3		Cakes, biscuits	0.3	
Lunch	25.8	28		26.2	27		22.9	26		25.2	29		23.9	29		27.1	29
Sausages*	1.1		Sausages*	1.4		Sausage dishes	0.9		Pies, pizza, toasted sandwiches*	1.3		Pies, pizza, toasted sandwiches*	1.8		Pies, pizza, toasted sandwiches*	1.7	
Pies, pizza, toasted sandwiches*	0.8		Pies, pizza, toasted sandwiches*	0.9		Pies, pizza, toasted sandwiches*	0.8		Sausage dishes	1.1		Sauces, dressings*	1.1		Sugar-sweetened beverages	1.0	
Sausage dishes	0.8		Sweetened beverages	0.6		Sauces, dressings*	0.6		Sausages*	0.8		Sugar-sweetened beverages	0.8		Sauces, dressings*	0.8	
Dinner	30.7	34		34.9	34		30.7	31		33.0	33		31.0	33		34.2	33
Pies, pizza, toasted sandwiches*	1.9		Pies, pizza, toasted sandwiches*	2.5		Pies, pizza, toasted sandwiches*	2.3		Pies, pizza, toasted sandwiches*	3.0		Pies, pizza, toasted sandwiches*	2.4		Pies, pizza, toasted sandwiches*	3.0	
Sugar-sweetened beverages	1.5		Sugar-sweetened beverages	1.9		Sugar-sweetened beverages	1.2		Sugar-sweetened beverages	1.8		Sauces, dressings	1.5		Sugar-sweetened beverages	1.8	
Potato products and dishes*	1.2		Sausages*	1.0		Sauces, dressings*	1.0		Potato products and dishes*	1.3		Sugar-sweetened beverages	1.5		Sausages*	1.3	
Snack/between meal	10.0	36		9.2	39		10.5	33		8.7	35		9.2	35		7.7	35
Cakes, biscuits	1.0		Cakes, biscuits	1.0		Cakes, biscuits	0.9		Cakes, biscuits	0.9		Cakes, biscuits	1.2		Cakes, biscuits	0.7	
Desserts, icecream	0.5		Pies, pizza, toasted sandwiches*	0.5		Sweets, chocolate	0.7		Sweets, chocolate	0.4		Pies, pizza, toasted sandwiches*	0.5		Pies, pizza, toasted sandwiches*	0.3	
Sweets, chocolate	0.5		Sweets, chocolate	0.5		Mixed fat spread*	0.3		Pies, pizza, toasted sandwiches*	0.3		Sweets, chocolate	0.4		Sugar-sweetened beverages	0.3	
Other	12.8	84		9.2	79		16.5	84		13.2	79		15.9	79		11.7	79
Sweets, chocolate	4.8		Sweets, chocolate	3.1		Cakes, biscuits	3.2		Sweets, chocolate	5.6		Sweets, chocolate	5.1		Sweets, chocolate	2.8	
Cakes, biscuits	2.2		Crisps, savoury snacks	1.6		Crisps, savoury snacks	1.7		Crisps, savoury snacks	1.9		Cakes, biscuits	2.6		Crisps, savoury snacks	2.2	
Crisps, savoury snacks	1.7		Cakes, biscuits	1.1		Desserts, icecream	1.0		Cakes, biscuits	1.3		Crisps, savoury snacks	2.0		Cakes, biscuits	1.6	
Beverage (mainly)	0.6	35		0.5	46		0.8	51		0.8	85		2.1	85		2.6	85
Sugar-sweetened beverages	0.1		Sugar-sweetened beverages	0.2		Cakes, biscuits	0.01		Sugar-sweetened beverages	0.4		Sugar-sweetened beverages	0.6		Sugar-sweetened beverages	0.7	
Sugar, syrup, honey	0.02		Sweets, chocolate	0.05		Cider, alcopops	0.01		Sweets, chocolate	0.02		Cider, alcopops	0.6		Beer	0.4	
Desserts, icecream	0.02		Dietary supplements	0.00		Desserts, icecream	0.01		Dietary supplements	0.01		Beer	0.1		Dietary supplements	0.4	

* Food group that contains both discretionary and non-discretionary foods
E, energy
Disc, discretionary
